# Spatio-temporal analysis of strawberry architecture: insights into the control of branching and inflorescence complexity

**DOI:** 10.1093/jxb/erad097

**Published:** 2023-03-26

**Authors:** Marc Labadie, Karine Guy, Marie-Noëlle Demené, Yves Caraglio, Gaetan Heidsieck, Amelia Gaston, Christophe Rothan, Yann Guédon, Christophe Pradal, Béatrice Denoyes

**Affiliations:** Univ. Bordeaux, INRAE, Biologie du Fruit et Pathologie, UMR 1332, F-33140, France; CIRAD, UMR AGAP Institut, F-34398 Montpellier, France; INVENIO, MIN de Brienne, 110 quai de Paludate, 33800 Bordeaux, France; INVENIO, MIN de Brienne, 110 quai de Paludate, 33800 Bordeaux, France; CIRAD, UMR AMAP and Université de Montpellier, 34398 Montpellier, France; Univ. Bordeaux, INRAE, Biologie du Fruit et Pathologie, UMR 1332, F-33140, France; CIRAD, UMR AGAP Institut, F-34398 Montpellier, France; Univ. Bordeaux, INRAE, Biologie du Fruit et Pathologie, UMR 1332, F-33140, France; Univ. Bordeaux, INRAE, Biologie du Fruit et Pathologie, UMR 1332, F-33140, France; CIRAD, UMR AGAP Institut, F-34398 Montpellier, France; CIRAD, UMR AGAP Institut, F-34398 Montpellier, France; Inria and LIRMM, Univ Montpellier, CNRS, Montpellier, France; Univ. Bordeaux, INRAE, Biologie du Fruit et Pathologie, UMR 1332, F-33140, France; CNB-CSIC Spain

**Keywords:** Branching, *Fragaria* × *ananassa*, hidden hybrid Markov/semi-Markov chain, multiscale tree graph, OpenAlea, plant architecture

## Abstract

Plant architecture plays a major role in flowering and therefore in crop yield. Attempts to visualize and analyse strawberry plant architecture have been few to date. Here, we developed open-source software combining two- and three-dimensional representations of plant development over time along with statistical methods to explore the variability in spatio-temporal development of plant architecture in cultivated strawberry. We applied this software to six seasonal strawberry varieties whose plants were exhaustively described monthly at the node scale. Results showed that the architectural pattern of the strawberry plant is characterized by a decrease of the module complexity between the zeroth-order module (primary crown) and higher-order modules (lateral branch crowns and extension crowns). Furthermore, for each variety, we could identify traits with a central role in determining yield, such as date of appearance and number of branches. By modeling the spatial organization of axillary meristem fate on the zeroth-order module using a hidden hybrid Markov/semi-Markov mathematical model, we further identified three zones with different probabilities of production of branch crowns, dormant buds, or stolons. This open-source software will be of value to the scientific community and breeders in studying the influence of environmental and genetic cues on strawberry architecture and yield.

## Teaser

*OpenAlea.Strawberry*open-source software, combining 2D and 3D representations and statistical methods, can be used to explore the genetic diversity and spatio-temporal evolution of plant architecture in cultivated strawberry.

## Introduction

Plant architecture is one of the most important agronomic traits that determine yield in crops (Mathan *et al*., 2016). It is under genetic control (Reinhardt and Kuhlemeier, 2002; Wang and Li, 2006) and influenced by environmental conditions (Teichmann and Muhr, 2015). The final architecture of the plant and subsequently the yield depend on the ontogenetic dynamics of the apical and axillary meristems.

Plant architecture is defined as the spatial organization of the plant (Hallé and Oldeman, 1970). In a typical plant architecture, the shoot apical meristem (SAM) establishes the primary axis of the plant by initiating a succession of vegetative entities named phytomers. A phytomer represents the basic unit of the plant axis and consists of an internode and a node with its attached leaf and its axillary bud (AXB). From this primary axis, the apical meristem from the AXB (the axillary meristem, AXM) will lead to the formation of axillary production (branch, flower, etc.). In sympodial growth, the SAM becomes a flower or an inflorescence, and growth is continued by one or more apical meristems from the AXBs, which repeat the process in secondary axes (Schmitz and Theres, 1999). While the axis is a succession of phytomers, the plant is a succession of axes, primary, secondary, tertiary, etc. In the plant, the module refers to a determinate axis (primary, secondary, tertiary, etc.) built up by a single SAM. The linear succession of modules constitutes an apparent axis (Barthélémy and Caraglio, 2007). Because of the repetition of these growth and branching processes, plants can be formally described through rooted multiscale tree graphs (MTG) whose vertices correspond to their constituent botanical entities and edges represent the physical connections between them (Godin and Caraglio, 1998). In this multiscale representation, the architecture can be described at phytomer and axis scales (Barthélémy and Caraglio, 2007) and visualized in two and three dimensions (Pradal *et al*., 2009; Pradal and Godin, 2020).

Perennial plants differ from herbaceous annual plants by their architecture, notably by their branching habit (Schmitz *et al*., 1999). Architectural studies have been developed for annuals (e.g. rice; Chen *et al*., 2020) and for woody perennials (Nicolini *et al*., 2003; Barthélémy *et al*., 2009). These studies have allowed the development of models for identifying ideotypes by correlating topology and yield capacity (e.g. in *Coffea*; Cilas *et al*., 2006). For the herbaceous perennial multiplying by seed model, tomato is the main species studied (e.g. Safaa *et al*., 2009). In contrast, the architecture of herbaceous perennial models multiplying by vegetative propagation, such as strawberry, remains poorly known.

**Fig. 1.**
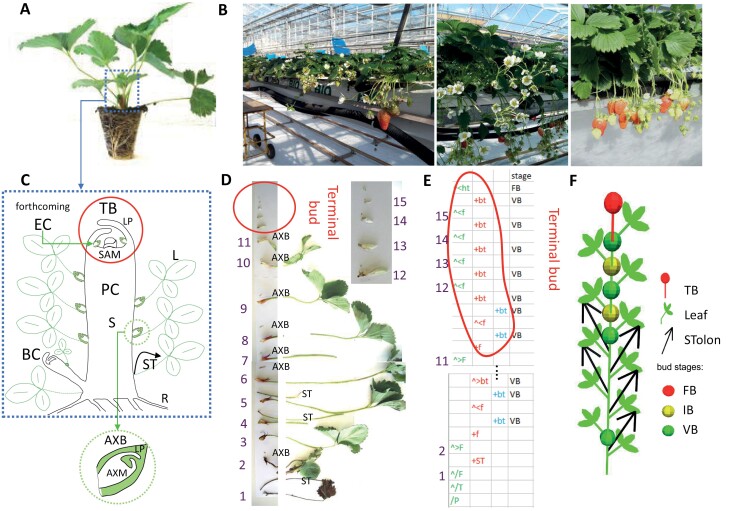
Tree-structured representation of a strawberry at planting. (A) Tray plant at planting. (B) Soil-less culture of strawberry in a glasshouse with a focus on flowering and fruiting plants. (C) Schematic representation of a primary crown (PC). PC includes stem (S), terminal bud (TB), and leaves with their axillary buds (AXB), which are dormant buds (non-extended lateral axes). A lateral branch crown (BC) is also represented. TB includes SAM and leaf primordia (LP). The uppermost AXB will produce the extension crown (EC). Vegetative AXB includes AXM and LP. L, leaf; R, root; ST, stolon. (D) Dissection of the plant with leaf order according to the rank of the node (numbered from 1 to 15). In the grey rectangle on the left are shown leaves with either stolon or AXB at their base. In the grey rectangle on the right are shown details of the dissection of the TB. (E) Multiscale tree graph (MTG) formalism of the plant with properties of stages. Colors represent the module order: green for order 0, red for order 1, and blue for order 2. Botanical entities as named in MTG: BT, bud; F, leaf; f, leaf primordium (leaf enclosed in the bud); FB, floral bud; ht, inflorescence enclosed in BT; HT: visible inflorescence (not represented in this plant); ST, stolon; VB: vegetative bud. ‘+’ indicates a branching link and ‘<’ a succession link. (F) 2D schematic representation of the plant generated by the *OpenAlea.Strawberry* package. Only the PC (zeroth-order module) is represented. The colored spheres indicate the non-visible/undeveloped higher-order modules (starting with ‘f’) of the AXB, as determined by the developmental stage of the AXM. FB, floral bud; IB, initiated bud; TB, terminal bud; VB, vegetative bud.

Cultivated octoploid strawberry (*Fragaria* × *annanassa*) is the main berry fruit crop in the *Rosaceae* family. Under production conditions, new tray plants (Fig. 1A), which are daughter plants obtained through vegetative propagation in a nursery, are planted each year. Fruit yield is determined by the intensity and duration of flowering (Fig. 1B). Following vegetative multiplication by a stolon, which is a specialized and highly elongated stem, SAM activity produces a primary axis, named the primary crown (PC) in strawberry (Fig. 1C). It consists of short internodes bearing adventitious roots, stipuled trifoliate leaves arranged in a rosette in spiral phyllotaxis of ⅖ (Savini *et al*., 2006), and AXBs containing AXMs, and is terminated by an inflorescence. Its development is sympodial: after its PC has stopped growing, the strawberry plant continues its vegetative growth from the highest AXM of the crown, which produces an axis called the extension crown (EC) (Fig. 1C) (Costes *et al*., 2014). The originality of the strawberry plant lies in the fate of the other AXMs along the module (Tenreira *et al*., 2017; Gaston *et al*., 2020) as they can either develop into lateral axes, named lateral branch crowns (BC) (Sugiyama *et al*., 2004), or into stolons, or stay dormant (Fig. 1C). Their fate, which is controlled by genetic and environmental factors (Tenreira *et al*., 2017; Andrés *et al*., 2021; Gaston *et al*., 2021), has a direct impact on plant and fruit yield. To date, nothing is known about the spatial organization of these structural components and their temporal interdependencies. Indeed, the various attempts to visualize strawberry architecture by Guttridge (1955) and more recently by Savini *et al*. (2006) and Massetani *et al*. (2011) could not determine the fate of the different meristems of a strawberry plant according to spatial and temporal patterns.

The objective of this work was to explore and identify the variability in spatio-temporal development of plant architecture among contrasting seasonal genotypes of cultivated strawberry. As strawberry plants are bushy and compact, we chose to dissect subsets of plants. The dissection was done at successive dates during the seasonal production in order to understand the architectural development. The plant architecture was fully described at the phytomer and module scales and then encoded in the MTG. In order to achieve our objective, we design a pipeline of methods including MTG-encoded plant description and various exploratory tools, visualization techniques, exploratory methods, and statistical modeling approach. The modeling approach relies on hidden hybrid Markov/semi-Markov (HHMSM) chains, a variant of hidden semi-Markov chains previously used to analyse branching patterns in many plant species (Guédon *et al*., 2022). Altogether, our results provide a new practical framework for strawberry architecture representation and analyses and, beyond, for modeling plant flowering and yield.

## Materials and methods

### Experimental protocol and datasets

#### Plant material and growth conditions

Five seasonal flowering varieties (Capriss, Ciflorette, Clery, Darselect, and Gariguette) and the advanced line Cir107 of cultivated octoploid ­strawberry (*Fragaria* × *ananassa*) were studied, as in Labadie *et al*. (2019). The plants of the six varieties used in the experiment were produced at a single location in a nursery in Douville. For the six varieties, runners bearing daughter plants emerged in mid-June from the mother plants. The unrooted daughter plants were taken from the runners at the end of July and kept at 4 °C in a climatic chamber for 1 week before being planted in trays containing a peat-based medium for rooting. For the six varieties, these plants, referred to as tray plants, were placed side by side outdoors under shade and thus subjected to the same environmental conditions (Supplementary Fig. S1 for temperature and global radiation conditions). To meet chilling requirements, which differed between genotypes (Supplementary Table S1), the tray plants were placed in a climate chamber at 2 °C at different dates in November until planting in December. All the genotypes were planted on 10 December 2014 except Ciflorette (4 December) in soil-less culture under tunnel in breeding ground-bags (Orgapin) of 10 liters with drip irrigation and fertilization at a minimal temperature of 8 °C. The tunnel was opened laterally when temperatures were above 18 °C.

#### Architectural and morphological data

**Fig. 2.**
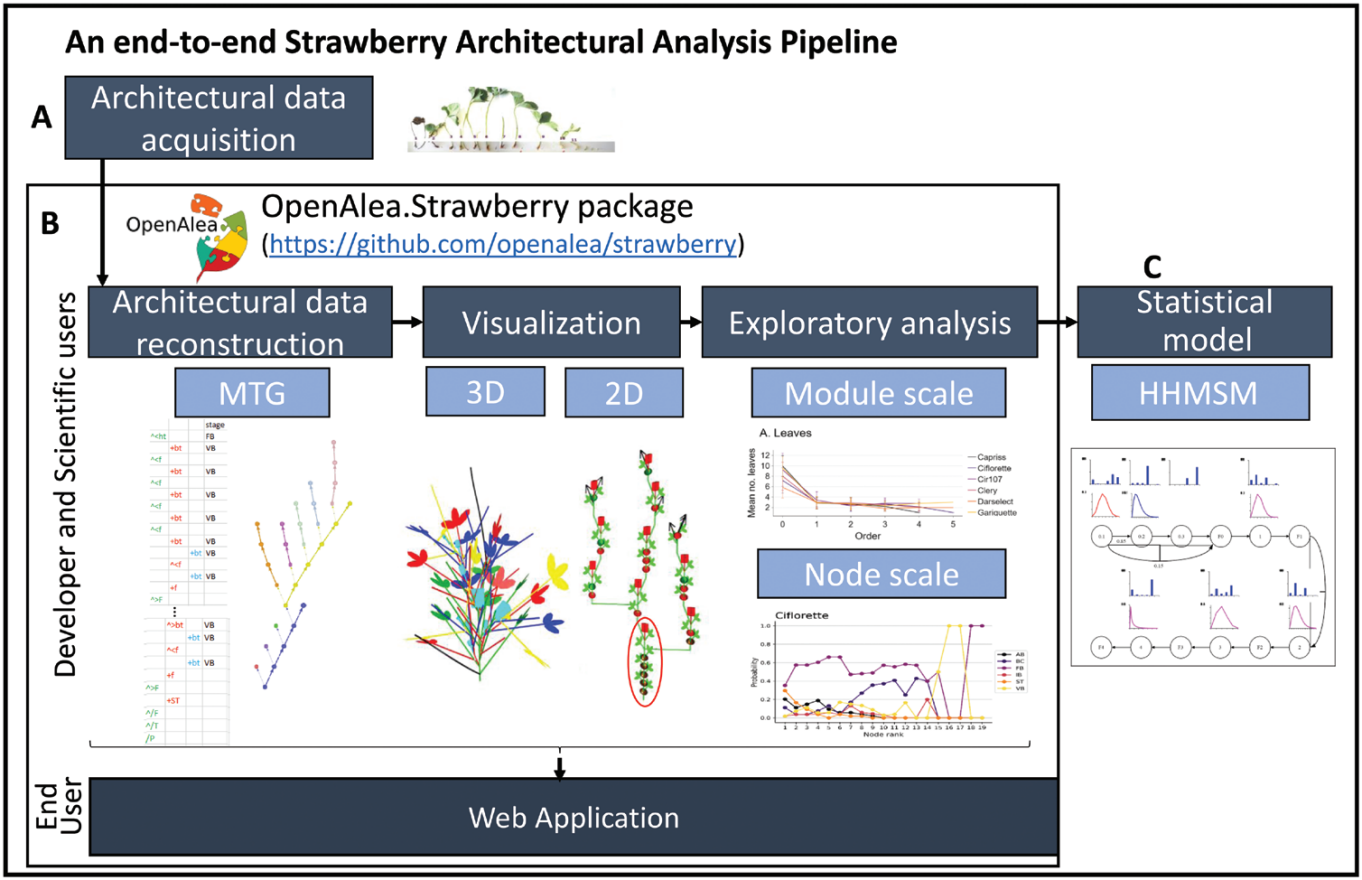
An end-to-end pipeline for the architectural analysis of strawberry with the *OpenAlea.Strawberry* package developed in Python on the OpenAlea platform. (A) Architectural data from the strawberry plants were acquired after dissection. Production of axillary buds and the stage of all terminal meristems were described. (B) Data were transposed in MTG formalism. The pipeline allows users to visualize architectural data in 3D and 2D and explore data at different scales: plant, module or node scale. In the 2D visualization, an example of a module (here the zero-order module) composed of a succession of phytomers is ringed in red. The succession of modules is represented by a diagonal division between the modules at an angle of 30°, while the branching is represented by a horizontal division at an angle of 90°. BC, lateral branch crown; FB, floral bud; IB, initiated bud; ST, stolon; TB, terminal bud; VB, vegetative bud. (C) In addition to the strawberry package, a statistical Markov analysis model (HHMSM model) was developed to characterize homogeneous zones of axillary production along apparent axes.

The architecture of nine plants per genotype was described approximately once per month at the same date in order to characterize the spatio-temporal development of the six genotypes from planting (Fig. 1D) and to model their architecture (Fig. 1E, F). The overall strategy is presented in Fig. 2A. It should be noted that the architecture was described exhaustively at the phytomer, module, and plant scale, and the observation protocol consisted of plant dissection. This experimental protocol indeed favors the topological dimension with respect to the temporal dimension. The plant architecture was encoded in the MTG format (see Figs 1E, 2B and Supplementary Table S2 for details regarding the specification of this MTG). This defines the types of botanical entity (e.g. plant, axis, phytomer), their attributes, and the type of relationship between elements (succession or branching). Each module contains a succession of phytomers with their type of axillary production (Fig. 2B). The order of a module corresponds to its branching order, i.e. the number of successive branching with zeroth-order (order 0) module for the PC. We studied the three following types of axillary production: a BC, which is a lateral extended axis with expanded leaves; a stolon, which is characterized by an elongated stem even at early stages of development; and a dormant bud. Dormant buds are non-extended lateral axes in which leaf primordia are contained within the bud (Fig. 1C). For dormant buds, we described the developmental stage of their apical meristem (AXM) by binocular observation. The bud stage (Jahn and Dana, 1970) was classed into four categories: (i) aborted (meristem showing necrosis), (ii) vegetative (meristem dome is relatively flat), (iii) initiated (meristem dome raises above the developing stipules), and (iv) floral (from sepal development to emergence of inflorescence). The total number of flowers of each inflorescence was counted when the inflorescence emerges. The EC (Fig. 1C) was not included in the description of axillary production but was extracted when analyses were performed on the apparent axis.

#### Extraction of data at the module scale

To study the occurrence with time of successive branching modules according to the genotype, the frequency distribution of module orders was extracted from the MTG for six successive dates of observation (mid-December, early January, mid-February, early March, early April, early June) and for each genotype. Modules considered for the statistical analysis had up to the fourth order. This is due to the absence of fifth-order modules for Clery, Capriss, and Cir107 and their low frequencies for the three other genotypes (one for Gariguette, three for Ciflorette, and one for Darselect). The only exception is the analysis of the dates of appearance of successive modules according to their orders. To study the composition of each module according to its order and genotype, the frequency distribution of the number of phytomers, number of flowers of the terminal inflorescence, and number of stolons was extracted for each module order and genotype by pooling all observation dates. These frequency distributions were explored and compared using standard statistical methods (e.g. ANOVA on ranks with post-hoc tests for count variables) and graphical representations (Fig. 2B).

#### Extraction of data at the node scale

We investigated the homogeneous zone of the axillary production along the zeroth-order module and their sympodial axes using a HHMSM model described hereafter. Sequences of axillary production were extracted at the node scale along the apparent axis according to the rank of the phytomer along each module (Supplementary Table S3). The sample size was between 107 sequences of cumulative length 909 for Darselect and 212 sequences of cumulative length 1707 for Capriss.

### Three-dimensional and two-dimensional visual exploration of the architecture

Two types of architectural visualization were designed: (i) a three dimensional (3D) canopy exploration and (ii) a two-dimensional (2D) individual schematic representation based on the MTG within the *OpenAlea.Strawberry* Python package (see ‘Results’).

A schematic 3D visualization of the canopy (Fig. 2B) highlights the 3D spatial organization of the plant, including the phyllotaxy. Vegetative development was represented using a color scale based on module order: zeroth order (order 0, green), first order (order 1, red), second order (order 2, blue), third order (order 3, yellow), and fourth order (order 4, purple). The timing and intensity of flowering were indicated by modulating the size and shape of the light blue boxes representing the inflorescences (Fig. 2B).

The 2D schematic visualization (Figs 1F, 2B) of the plant architecture is inspired by botanical sketches and highlights the sympodial branching as well as the different structures of the plant. The same representation was used for the axillary buds in 3D. Inflorescences were visualized as a red rectangle. Axillary buds were represented by colored spheres according to their SAM stage. This 2D visualization allows summarized architectural patterns not visible in 3D such as type of axillary production, which is not apparent in real-looking 3D visualization due to the bushy habit of the strawberry and the apparent axis as a linear succession of modules.

#### Standardization procedure for selecting the most central individual for 2D schematic representation

Regarding the potential high structural complexity of the architecture and the small sample size, we chose to extract a representative individual for each observation date and each genotype. To this end, we ordered the nine described plants within each sample from the most central to the most outlying using a robust standardized distance $d_{i}$ based on a few global variables $\left(x_{i,1\;},...,\;x_{i,j\;},...\right)$ (e.g. number of flowers, number of leaves, number of stolons, maximum module order and number of BC and EC):


$$\begin{array}{l} d_{i}=\underset{j}{\sum }\;\frac{\left|x_{i,\;j}-\;\underline{x_{j}}\right|}{MAD_{j}} \end{array}$$

where $MAD_{j}=\sum_{i}\left|x_{i,\;j}-\;\underline{x_{j}}\;\right|$ is the mean absolute deviation from the median $\underline{x_{j}}$ for the variable *j*.

### Hidden hybrid Markov/semi-Markov chains to model axillary production along apparent axis

The objective of our statistical modeling approach was to highlight a spatial organization of axillary production. We based our statistical model on a HHMSM model (Durand *et al*., 2005; Guédon, 2005) (Fig. 2C). The sequences used for inferring the model were apparent axes extracted at the node scale from architectural data with two variables: the module order and its axillary production. Module order is an instrumental variable to separate successive modules within the model. To this end, a HHMSM model was built for each genotype. A HHMSM chain is a two-scale segmentation model whose aim is to segment apparent axes in successive modules and within zeroth-order modules in successive homogeneous axillary production zones. The model is defined by its states (Markovian and semi-Markovian), transition probabilities between states to model the succession of zones along the apparent axes, occupancy distributions to model the zone length in number of phytomers, and categorical observation distributions to model the axillary production within a zone. Occupancy and categorical distributions are attached only to semi-Markovian states.

Hybrid Markov/semi-Markov chains can be defined as follows. Let $\left\{S_{t}\right\}$ be a hybrid Markov/semi-Markov chain with finite-state space {0,…,J−1}. A *J*-state hybrid Markov/semi-Markov chain $\left\{S_{t}\right\}$ is defined by the following parameters:

- initial probabilities $\pi_{j}=P(S_{1}=j)$ with $\sum_{j}\pi_{j}=1$;
- transition probabilities
  - semi-Markovian state *i*: for each $j\neq i,\;p_{ij}=P(S_{t}=j|S_{t}\neq i,S_{t-1}=i)$ with $\sum_{j\neq i}p_{ij}=1$ and $p_{ii}=0$ by convention,
  - Markovian state *i*: $p_{ij}=P\left(S_{t-1}=i\right)$ with $\sum_{j}p_{ij}=1$.

It should be noted that absorbing states (a state is said to be absorbing if, after entering this state, it is impossible to leave it) are necessarily Markovian.

An explicit occupancy distribution is attached to each semi-Markovian state:


$$\begin{array}{l} d_{j}\left(u\right)=P\left(S_{t+1}=j,S_{t}\neq j\right),\;u=1,2,\ldots \end{array}$$

Since the process starts out at *t*=1 in a given entering state *j*, the following relation is verified:


$$\begin{array}{l} P\left(S_{t}\neq j,S_{t-v}=j,v=1,\ldots ,t\right)=d_{j}\left(t\right)\pi_{j}. \end{array}$$

We define as possible parametric state occupancy distributions binomial distributions, Poisson distributions, and negative binomial distributions with an additional shift parameter *d* (*d*≥1), which defines the minimum sojourn time in a given state.

The binomial distribution with parameters *d*, *n*, and *p*(*q*=1−*p*), B(*d*, *n*, *p*) where 0≤*p*≤1, is defined by:


$$\begin{array}{l} d_{j}\left(u\right)=\left(\frac{n-d}{u-d}\right)p^{u-d}q^{n-u},\;u=d,d+1,\ldots ,n. \end{array}$$

The Poisson distribution with parameters *d* and *λ*, P(*d*, *λ*), where *λ* is a real number (λ>0), is defined by:


$$\begin{array}{l} d_{j}\left(u\right)=\frac{e^{-\lambda }\lambda^{u-d}}{\left(u-d\right)!},\;u=d,d+1,\ldots \end{array}$$

The negative binomial distribution with parameters *d*, *r* and *p*, NB(*d*, *r*, *p*), where *r* is a real number (*r*>0) and 0<*p*≤1, is defined by:


$$\begin{array}{l} d_{j}\left(u\right)=\left(\frac{u-d+r-1}{r-1}\right)p^{r}q^{u-d},\;u=d,d+1,\ldots \end{array}$$

A HHMSM chain (Guédon, 2005) can be viewed as a pair of stochastic processes $\left\{S_{t},X_{t}\right\}$ where the ‘output’ process $\left\{X_{t}\right\}$ is related to the ‘state’ process $\left\{S_{t}\right\}$, which is a finite-state hybrid Markov/semi-Markov chain, by a probabilistic function or mapping denoted by *f* (hence $X_{t}=f(S_{t})$). Since the mapping *f* is such that a given output may be observed in different states, the state process $\left\{S_{t}\right\}$ is not observable directly but only indirectly through the output process $\left\{X_{t}\right\}$. This output process $\left\{X_{t}\right\}$ is related to the hybrid Markov/semi-Markov chain $\left\{S_{t}\right\}$ by the observation (or emission) probabilities $b_{j}(y)=P\left(S_{t}=j\right)$. The definition of the categorical observation distributions expresses the assumption that the output process at time *t* depends only on the underlying hybrid Markov/semi-Markov chain at time *t*.

The maximum likelihood estimation of the parameters of a HHMSM chain requires an iterative optimization technique, which is an application of the expectation-maximization algorithm. Once a HHMSM chain has been estimated, the most probable state sequence can be ­computed for each observed sequence using the so-called Viterbi algorithm; see Guédon (2003, 2005, 2007) for the statistical methods for HHMSM models. In our application context, the most probable state sequence corresponding to the zeroth-order module can be interpreted as the optimal segmentation of the corresponding observed sequence into successive axillary production zones.

**Fig. 3.**
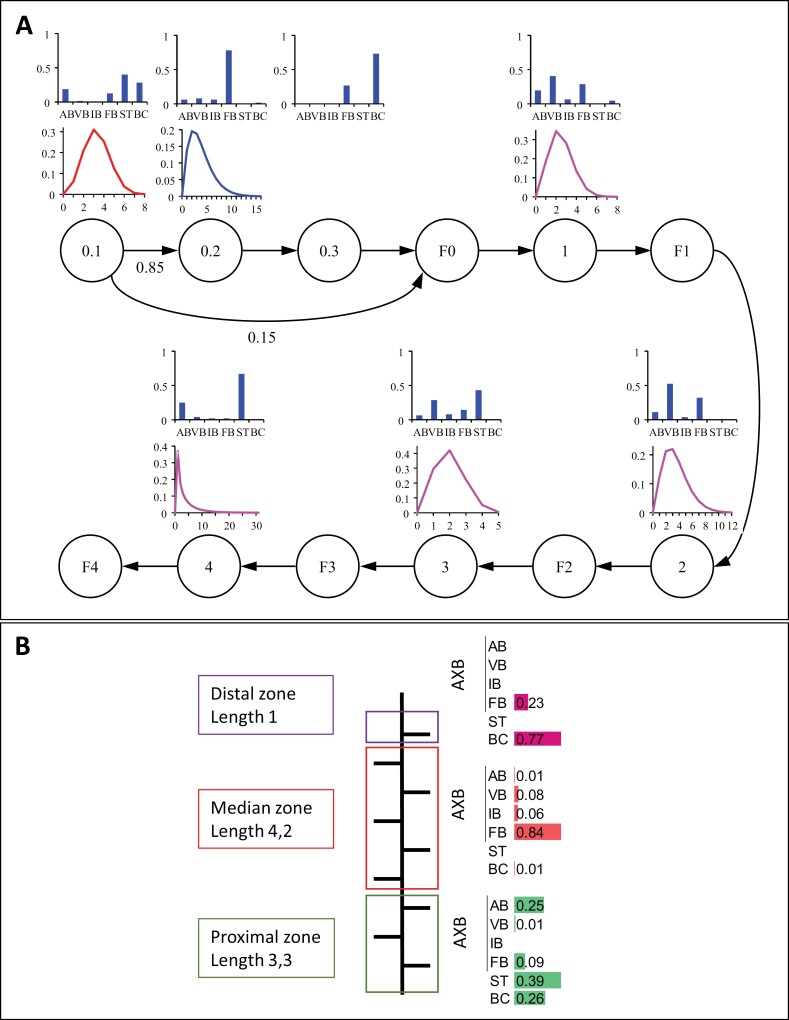
Schematic representations of the hidden hybrid Markov/semi-Markov chain estimated for Ciflorette. (A) Hidden hybrid Markov/semi-Markov chain and its parameters values. Each vertex represents either a Markovian or a semi-Markovian state. Markovian states correspond to terminal inflorescences with their module orders (states F0, F1, F2, F3, and F4). Semi-Markovian states represent zones within the zeroth-order module (0.1: proximal zone, 0.2: median zone, 0.3: distal zone) and module orders (1, 2, 3, 4). Transition probabilities are represented by arrows between states (edge). Transition probabilities are noted nearby when <1. The observation distributions, which represent the zone composition (proportions of axillary production), and the occupancy distributions, which represent the zone length in number of nodes, are attached to each state and are at the bottom of the corresponding vertex. *x*-axis: zone composition; *y*-axis: probability mass. For occupancy distribution: *x*-axis: number of successive nodes (# nodes); *y*-axis: probability mass. (B) Hidden hybrid Markov/semi-Markov summarized on plant architecture for zeroth-order module. Each zone is represented by a color box: green for proximal zone, red for median zone, and purple for distal zone. Each zone was characterized by the mean length zone (mean no. phytomers) and the non-generated observation distribution of axillary production colored in data bars. Axillary production was axillary buds (AXB) determined by the developmental stage of their AXM. AB, aborted bud; BC, lateral branch crown; IB, initiated bud; FB, floral bud; ST, stolon; VB, vegetative bud.

In our context, the zeroth-order module is modeled as a chain of consecutive semi-Markovian states to decompose it in successive axillary production zones (states 0.1, 0.2, and 0.3 in Fig. 3A; distribution of axillary production along the zeroth-order module in Fig. 3B). Higher order modules are modeled by an alternation of Markovian states corresponding to terminal inflorescences (states F0, F1, F2, F3, and F4 represented in Fig. 3A for the Ciflorette model) followed by a semi-Markovian state to represent homogeneous axillary production zone within a module (states 1, 2, 3, and 4 are semi-Markovian states in Fig. 3A).

For zeroth-order modules, the almost deterministic succession of states was used to assess the number of zones. We thus expected that the states stayed ordered and that the probabilities of skipping states were low as a byproduct of the iterative estimation procedure of a HHMSM model.

The maximum likelihood estimation procedure computes the parameters of the model, i.e. the probability of transition between two zones, the distribution of axillary production for a given zone, and numbers of phytomers of a given zone. To this end, it uses an iterative optimization technique, which is an application of the expectation-maximization algorithm (Ephraim and Merhav, 2002). Once a HHMSM model has been estimated, the most probable state sequence can be computed for each observed sequence using the so-called Viterbi algorithm (Guédon, 2003, 2005, 2007). In our context, the most probable state sequence corresponding to the zeroth-order module can be interpreted as the optimal segmentation of the corresponding observed sequence into successive axillary production zones.

On the basis of the optimal segmentations of the observed sequences, model parameters were extracted for each genotype (Renton *et al*., 2006) in order to compare zeroth-order modules between genotypes on the basis of the global model estimated on the pooled sample.

Moreover, to have a global view of the dissimilarities between the axillary production patterns of the genotypes, we first computed the matrix of pairwise dissimilarities between HHMSM chains using the Kullback–Leibler divergence, and then applied a hierarchical clustering approach on this matrix (Guédon *et al*., 2003). The dissimilarity measure is computed on generated axillary production sequences of fixed length, systematically starting with zeroth-order modules and ending with fourth-order modules. We also applied this approach to the comparison of the axillary production patterns of the genotypes restricted to the zeroth-order modules.

## Results

### Spatio-temporal description of strawberry plant architecture using 3D and 2D representations

The strawberry plant has a compact structure (Fig. 1A), and therefore requires dissection to fully decipher its architecture. The terminal bud is located at the upper end of the PC and comprises the SAM and leaf primordia (Fig. 1C). The terminal bud is surrounded by the petioles of developed leaves and is thus not visible on intact plants. The AXBs are located at the axil of each leaf and are only visible when leaves are removed. As shown in Fig. 1D, the AXB produces either a stolon or a BC or remains dormant (a non-extended axis). A BC is a lateral axis comprising expanded leaves with an AXM at their axil and also an inflorescence under favorable conditions. Dissection of the AXB allows the characterization of its developmental stage according to the stage of its apical meristem (AXM) (Jahn and Dana, 1970; Gaston *et al*., 2021; see ‘Materials and methods’). The meristem of a vegetative bud is vegetative, the meristem of an initiated bud is at the first stages of floral initiation (Fig. 1C), while the meristem of a flowering bud is at more advanced stages of flower and inflorescence development. Strawberry plant development is therefore viewed as a succession of incremental order modules (Fig. 1E). The zeroth-order module is the PC. Along this module, AXBs will produce either first-order modules (BCs or ECs) or stolons, as shown in the 2D representation of the plant (Fig. 1F). BCs and ECs will produce higher order modules and stolons, etc.

We reasoned that spatio-temporal variations in module-orders in cultivated strawberry (*Fragaria* × *ananassa*) should result in a diversity of plant architecture and flowering patterns and ultimately in yield patterns. To test this hypothesis, we studied six strawberry genotypes contrasting in plant architecture and fruit yield. The architecture was described exhaustively at the phytomer, module, and plant scale levels using an experimental protocol that favors the topological dimension with respect to the temporal dimension. The plant architecture was encoded in the MTG format (Fig. 1E; Supplementary Table S2 for details regarding the specification of this MTG). This allowed us to obtain 2D (Fig. 1F) and 3D representations of the plant.

**Fig. 4.**
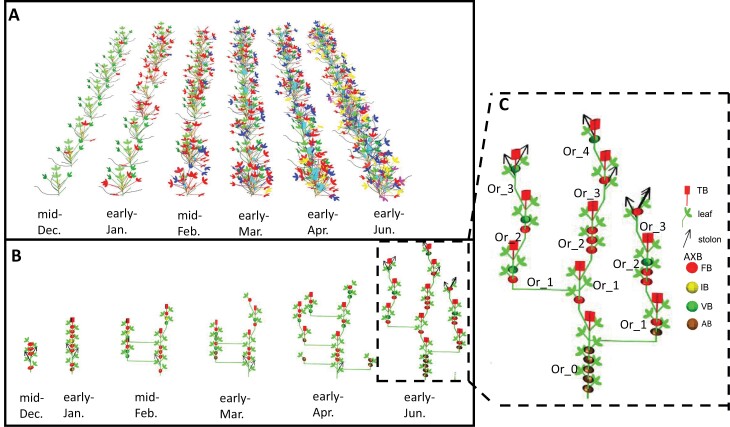
Spatio-temporal architecture of strawberry plant using 3D and 2D representations. (A) 3D representation of the nine plants of the genotype Ciflorette during the seasonal production. The seasonal production is represented by six successive dates of observation. Colors represent module orders: green for order 0, red for order 1, blue for order 2, yellow for order 3, and purple for order 4. The size of the light blue boxes representing the inflorescence is proportional to the number of open flowers. (B) 2D representation of the genotype Ciflorette during seasonal production. The spatio-temporal architecture of Ciflorette is summarized by the representation of the most central individual for each date of observation. The representation of the development of a given genotype is discontinuous between two dates because the acquisition of the architecture requires dissection and is destructive. At each date, a new plant of the same genotype is represented. Each organ is represented by a geometrical shape: inflorescence, red box; phytomer (internode + petiole + three leaflets), green cylinder terminated by three green discs; stolon, black arrow; axillary bud (AXB), a sphere colored according to the stage of the terminal meristem (green, yellow, red or brown for respectively vegetative, initiated, floral or aborted/dried stage). Lateral branch crown and extension crown are represented by horizontal or oblique shifts, respectively. Apparent axes are materialized by the succession of the extension module order from zeroth-order module or from a lateral branch crown. (C) 2D representation of the most central plant of the genotype Ciflorette at the last date of observation. Module order rank is indicated by the number on the side of each module. At this last observation date, the dry dormant buds observed were considered as aborted buds. They were mainly located on the zero-order module. AB, aborted bud; IB, initiated bud; FB, floral bud; TB, terminal bud; VB, vegetative bud.

The 3D visualization of the canopy enabled the visual exploration of the reconstructed plants in order to determine the spatial organization, the flowering intensity, and the phenology of the different genotypes (Fig. 4A for Ciflorette; Supplementary Figs S2, S3 for all genotypes). We can observe that at planting (mid-December) all the Ciflorette individuals consisted of zeroth- and first-order (green and red, respectively) modules. Then, second-order (blue) modules appeared in mid-February. Third-order (yellow) and fourth-order (purple) modules appeared in early May. Strikingly, the nine individuals of each genotype at each successive date of observation show a homogeneous development, with similar module order distributions. When comparing all genotypes, we observed that they differed by their earliness of module development (Supplementary Fig. S2). For Gariguette, Clery, and Ciflorette, first-order modules appeared in mid-February while they appeared in early January for Capriss and Cir107 and in mid-December for Darselect. Second-order modules appeared in early March for Capriss, Gariguette, Clery, and Ciflorette, while they appeared in mid-February for Cir107 and Darselect. Third- and fourth-order modules appeared at the last date of observation (early June) except for Darselect, for which third-order modules appeared in early April. The six genotypes also showed marked differences in flowering intensity, which is reflected in the different sizes of the light blue boxes representing the number of flowers per inflorescence, clearly visible in 3D visualization without leaves (Supplementary Fig. S3). For Gariguette and Cir107, flowering was already intense for all plants in early March, while at this date flowering started for the four other genotypes.

The 2D representation of the spatio-temporal architecture is represented by the most central plant for each date of the six successive observations (Fig. 4B for Ciflorette; Supplementary Fig. S4 for all genotypes). This representation highlights the succession of incremental order modules (Fig. 4C), the spatio-temporal development of branching, and its variability between genotypes (Supplementary Fig. S4). AXB description was summarized by the stage of its meristem. As an example, along plant development, Ciflorette produced BCs in addition to its ECs (Fig. 4B, C). These branches occurred mainly in the proximal and distal zones of the PC (zeroth-order module). In this zeroth-order, AXBs of the median zone were dormant and already initiated at plantation; they did not develop into BCs during plant development and instead dried (rated as aborted). Comparison of 2D architectures between the six genotypes shows that they differ by their complexity and in particular by their branching behavior. This difference in branching leads to different numbers of BCs. In early June, Gariguette showed two apparent axes, while Ciflorette, Clery, and Darselect showed three and Capriss and Cir107 four (Supplementary Fig. S4). In addition, distribution of BCs along the zeroth-order module ­differed ­between genotypes, the three of Capriss and Cir107 being distributed from the bottom to the top while the single ones of Gariguette were at the top. However, the six genotypes were very similar with respect to the fate of the AXBs localized on the median zone of the zeroth-order since they were almost all initiated at plantation and stayed dormant or aborted during the experiment.

### Exploration of the architectural development of strawberry and its genetic modulation

**Fig. 5.**
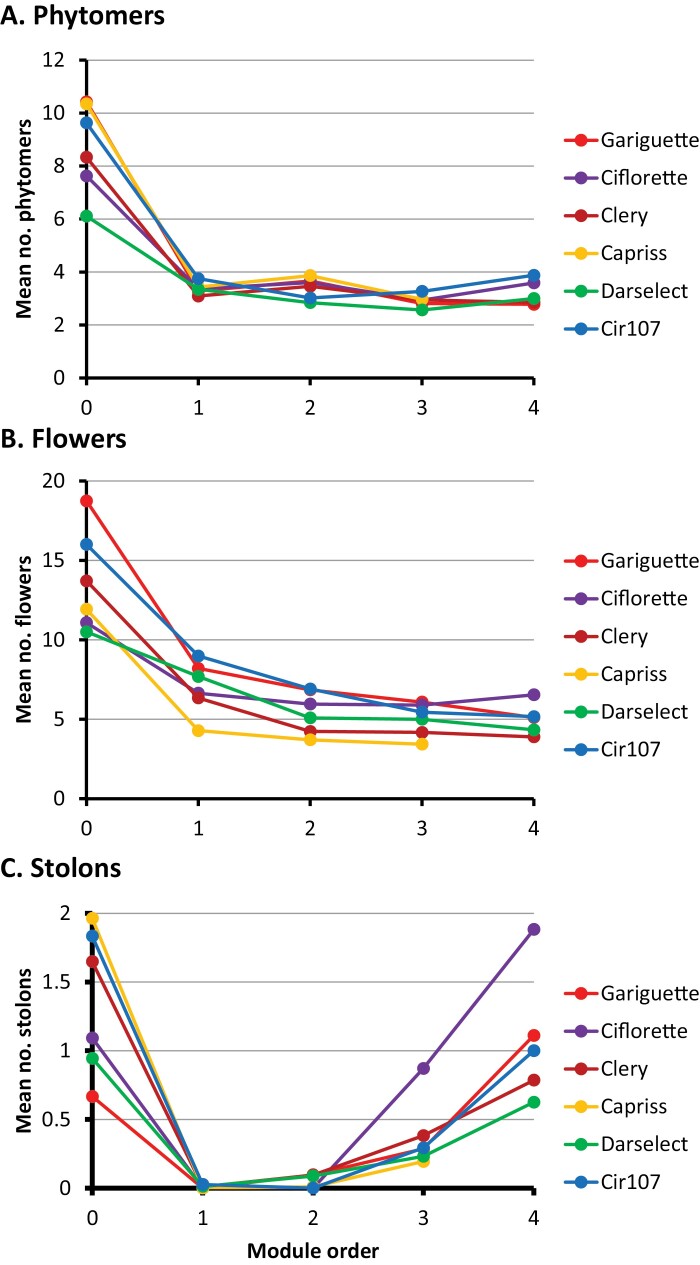
Mean number of phytomers (A), flowers (B), and stolons (C) as a function of module order for each of the six genotypes. Data represent means of nine plants per genotype.

Regardless of their differences, the strawberry genotypes share a common architectural pattern characterized by the following:

- A zeroth-order module with a far higher number of phytomers (6–10) than the higher-order modules (2–4) (Fig. 5A), the number of phytomers of the modules of order ≥1 being roughly constant for most of the genotypes (see the linear trends estimated for the number of phytomers as a function of the order for order ≥1 in Supplementary Table S4).
- A terminal inflorescence with a far higher number of flowers for the zeroth-order module (10–18) compared with higher-order modules (Fig. 5B), the number of flowers being most often slightly higher for first-order modules (4–8) compared with modules of order ≥2 (4–6) (see the linear trends estimated for the number of flowers as a function of the order for order ≥2 in Supplementary Table S4).
- The presence of stolons in zeroth-order modules, their absence in first-order modules, and their near absence in second-order modules, then the number of stolons per module increasing with the module order (Fig. 5C).

There are quantitative differences between genotypes, these being more pronounced for the zeroth-order modules and for flowering intensity (Fig. 5A–C). Concerning the vegetative development, the genotypes can be ordered in the following way according to the number of phytomers of the zeroth-order module (Fig. 5A; Supplementary Table S5): (Gariguette, Capriss, Cir107)>(Ciflorette, Clery)>Darselect. Concerning the complexity of the terminal inflorescence, in term of the number of flowers, Gariguette and Cir107 were the genotypes with the most complex inflorescences whatever the module order, while Capriss exhibited the least complex inflorescences (Fig. 5B; Supplementary Table S6). For the three other genotypes with intermediate inflorescence complexity (Ciflorette, Clery, and Darselect), the complexity varies according to the module order.

Concerning the stolons, the situation contrasts between zeroth-order modules and modules of order ≥3 (Fig. 5C; Supplementary Table S7). For instance, Capriss exhibited the highest number of stolons for the zeroth-order module and the lowest for third-order modules, while Ciflorette exhibited an intermediate number of stolons for the zeroth-order module but the highest number of stolons by far for third- and fourth-order modules.

Finally, concerning the number of modules of order ≥1, Capriss and Cir107 were the most bushy genotypes with the highest numbers of first- and second-order modules, while Gariguette, Clery and Darselect were the less bushy genotypes with the lowest numbers of first- and second-order modules, Ciflorette being intermediate between these two groups (Supplementary Table S3).

To assess the dates of occurrence of successive module orders (Table 1), we used high-quantile criteria (0.9-quantile) that rely on the whole distribution of module order (Supplementary Table S8 for details on the distribution of module order for successive date). We used this approach to take into account the small sample sizes (nine individuals per observation date for each genotype) and the non-robustness of the maximum criterion. It should be noted that the highest module order was relatively homogeneous between individuals of a given genotype for each successive observation date as illustrated by the 3D visualization of individuals in Supplementary Fig. S2. This statistical analysis also revealed differences between the six genotypes with regard to the timing of module orders. At planting (mid-December), all genotypes were composed of zeroth and first-order modules except Gariguette, for which the first module order occurred 1 month later (early January). Second-order modules occurred less than 1 month later for Cir107 (early January) and less than 2 months after for Capriss, Ciflorette, and Darselect (mid-February). It is noteworthy that second-order modules occurred late for Clery and Gariguette (mid-March). Third-order modules occurred earlier for Darselect (mid-March) than for the five other genotypes (early June). At the last date of observation, the highest module order was 3 for Capriss and Cir107 and 4 for Gariguette, Clery, Ciflorette, and Darselect (Table 1). Overall, these results suggest differences in module occurrence along the culture according to genotypes.

**Table 1.** Occurrence of module order for each genotype and successive dates of observation

	Capriss	Ciflorette	Cir107	Clery	Darselect	Gariguette
Mid-Dec	1	1	1	1	1	0
Early Jan	1	1	2	1	1	1
Mid-Feb	2	2	2	1	2	1
Early Mar	2	2	2	2	3	2
Early Apr	2	2	2	2	3	2
Early Jun	3	3 and 4	3	3 and 4	4	3 and 4

The highest module order was extracted from the frequency distribution of module orders with quantile 0.9.

### Three well-differentiated successive zones in zeroth-order module based on axillary production using a mathematical model

**Fig. 6.**
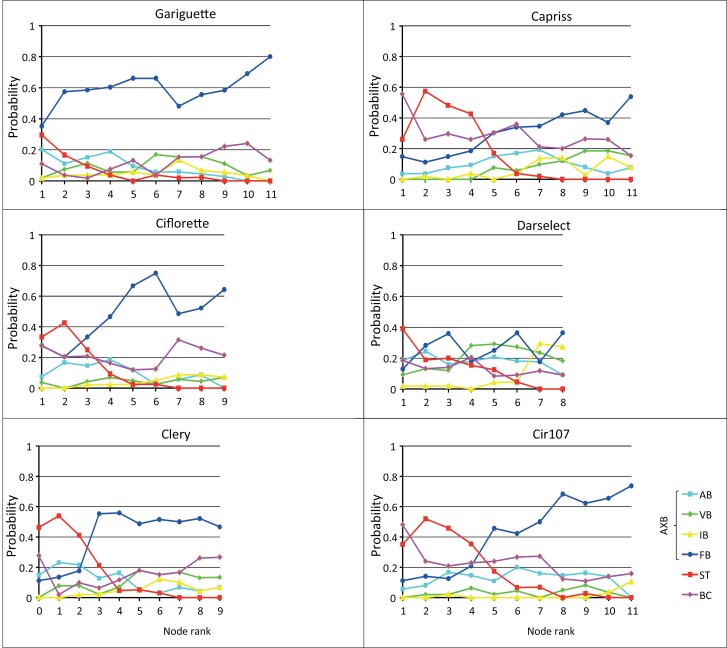
Probabilities of the axillary production as a function of the node rank for Gariguette, Ciflorette, Clery, Capriss, Darselect, and Cir107. The node rank is ordered from the base to the top of the zeroth-order module without the last node bearing the extension crown. Axillary production was axillary buds (AXB) determined by the developmental stage of their AXM. AB, aborted bud; BC, lateral branched crown; IB, initiated bud; FB, floral bud; ST, stolon, VB, vegetative bud.

The empirical probabilities of the axillary production were extracted from the observed sequences synchronized from the top or from the base along the zeroth-order module. These empirical probabilities show a proximal stolon zone followed by a flowering bud median zone when extracted from both the base and the top except for Gariguette (Fig. 6 and Supplementary Fig. S5, respectively). A distal short lateral BC zone (always a single phytomer) is observed when empirical probabilities were extracted from the top (Supplementary Fig. S5) but with a lower probability for Darselect (0.35) compared with the five other varieties (>0.60) (Table 2). We thus assumed that three well-differentiated successive zones (semi-Markovian states: 0.1, 0.2, 0.3) can be identified in the zeroth-order module until F0 Markovian state (i.e. inflorescence of the zeroth-order module) for all genotypes except Gariguette. For Gariguette, we assumed that only two well-differentiated successive zones could be identified (the three zones assumption was tested but, in this case, the iterative estimation algorithm of the HHMSM chain led to two first zones in parallel with rather similar compositions in terms of axillary production). A HHMSM chain was built for each genotype (Ciflorette in Fig. 3 and all genotypes in Supplementary Fig. S6; Tables 2, 3).

**Table 2.** Proximal, median and distal zones of the zeroth-order module for the six genotypes: lengths (number of leaves) and axillary production (as a percentage)

	Proximal zone							Median zone							Distal zone						
		Axillary production							Axillary production							Axillary production					
		AXB							AXB							AXB					
	Mean length	AB	VB	IB	FB	ST	BC	Mean length	AB	VB	IB	FB	ST	BC	Mean length	AB	VB	IB	FB	ST	BC
Gariguette								9	0.1	0.11	0.05	0.6	0.08	0.06	1.1				0.37		0.63
Ciflorette	3.3	0.18	0.02		0.12	0.4	0.28	3.9	0.06	0.08	0.06	0.78		0.02	1				0.27		0.73
Clery	3.5	0.24	0.03		0.13	0.49	0.11	5.1	0.04	0.17	0.08	0.7		0.01	1				0.19		0.81
Capriss	4.9	0.06		0.01	0.12	0.4	0.41	4.1	0.16	0.16	0.13	0.47		0.08	1	0.04			0.3		0.66
Cir107	4.2	0.04			0.11	0.52	0.33	5.7	0.2	0.06	0.01	0.62		0.11	1				0.02		0.98
Darselect	3.4	0.03	0.15		0.23	0.42	0.17	4.2	0.32	0.25	0.07	0.27	0.02	0.07	1	0.13		0.1	0.42		0.35

Axillary production was axillary buds (AXB) determined by the developmental stage of their AXM. AB, aborted bud; BC, lateral branch crown; FB, floral bud; IB, initiated bud; ST, stolon; VB, vegetative bud.

**Table 3.** Probabilities of skipping zones in zeroth-order modules

	Prox	Prox + Med	Med	Dist	Med + Dist
Gariguette					
Ciflorette	0.17				0.15
Clery	0.01	0.04	0.23		0.07
Capriss					0.02
Cir107	0.08	0.09			0.05
Darselect	0.36				0.62

Empty cells: For Gariguette, the proximal zone does not exist; otherwise, the probability is zero. Prox, proximal; Med, median; Dist, distal.

A global model was also built on the basis of the pooled sample with three zones for zeroth-order modules from which zone lengths and compositions were extracted for each genotype on the basis of the optimal segmentation in zones (Supplementary Table S9). This global model shows that the three successive zones in zeroth-order modules (proximal stolon zone, median bud zone, and distal short BC zone) are reasonably shared by all the genotypes. We obtained high percentages of match between the segmentations in three zones obtained using the global model and the genotype-specific model for all the genotypes (97% and 65.2%, respectively; Gariguette was excluded from the analysis). This global model also shows that both the probability of skipping the proximal zone is the highest and the probability of observing stolons in this proximal zone is the lowest for Gariguette (Supplementary Tables S9, S10). This is consistent with the impossibility of identifying this zone on the basis of the sole Gariguette sample.

We also built models with one more zone at the base of zeroth-order modules for Capriss and Cir107 consistently with the high empirical probability of observing a lateral branching at the base of zeroth-order modules for these two genotypes (Supplementary Fig. S5). This short zone was modeled by a Markovian state and confirms the specificity of Capriss and Cir107 regarding this pattern (Supplementary Fig. S7; Supplementary Table S11), which explains their bushy habit with far more first- and second-order modules compared with the other genotypes (Supplementary Table S3).

### Axillary meristem fate depends on the module order

Higher-order modules were modeled with only one homogeneous zone. We observed and quantified that first- and second-order modules contain predominantly vegetative buds and flowering buds for all genotypes (Supplementary Table S12; Fig. 3A for Ciflorette). From third-order modules, we showed an increase in the proportion of stolons, depending on the genotypes, at the expense of the proportion of flowering buds (Fig. 3A; Supplementary Table S12).

The transition probability evaluated the AXM fate of two successive buds in modules of order superior to zeroth-order (≥1) (Supplementary Table S13). The probability close to 1 for the transition BC→EC suggests that BCs are systematically ­followed by an EC, in the last phytomer of the module. Moreover, the transition probability leading to stolon (Supplementary Table S14) shows that stolons are rarely preceded by another axillary production in the module order ≥1. In summary, when lateral branching is observed in module orders superior to zeroth-order (86 BCs for top modules versus 1053 ECs), BCs were at the top and stolons were at the base of the module.

### Global comparison of genotypes

**Fig. 7.**
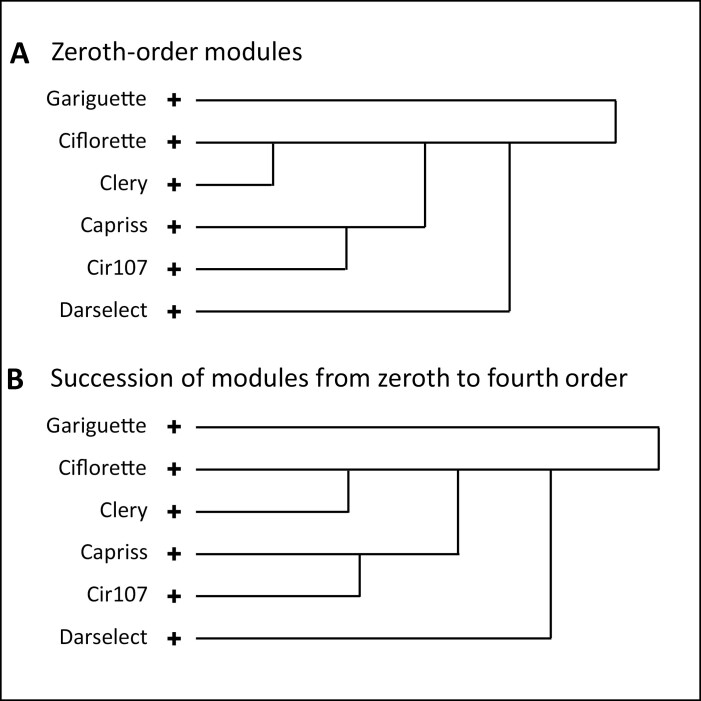
Hierarchical clustering dendrogram of axillary production patterns of the six varieties on the basis of matrix of pairwise dissimilarity measures between models. The matrix of pairwise dissimilarity was calculated on the basis of Kullback–Leibler divergences between estimated hidden hybrid Markov/semi-Markov chains for zeroth-order modules (A) and a succession of modules from zeroth to fourth order (B). The length of the edge between two genotypes in each dendrogram represent the distance or dissimilarity between these genotypes. The ‘+’ sign indicates the terminal node of the clustering tree.

Dissimilarity measures between models corresponding to succession of modules from zeroth to fourth order estimated for each genotype (Table 2) and between models restricted to zeroth-order modules were computed. The clustering of genotypes (Fig. 7) on the basis of a matrix of pairwise dissimilarity measures between models mainly reflects the difference between zeroth-order modules (Table 2):

- Gariguette, with no well-defined proximal stolon zone;
- Ciflorette and Clery, with the highest proportion of flowering bud in the median bud zone;
- Capriss and Cir107, with far more BC at the base of the zeroth-order module;
- Darselect, with few flowering bud in the median zone and few BC in the distal zone.

It should be noted that this clustering is consistent with the ordering of genotypes according to the number of phytomers of the zeroth-order module (Fig. 5; Supplementary Table S5) with (Gariguette, Capriss, Cir107)>(Ciflorette, Clery)>Darselect and with the far more bushy habit of Capriss and Cir107 with respect to the other genotypes (Supplementary Table S3). A 2D schematic representation of most central individuals (Supplementary Fig. S4) highlights the delayed appearance of first-order modules in Gariguette and the more basal branching of Capriss and Cir107, and to a lesser extent of Ciflorette.

### Open-source software and web application for 3D visualization and architectural analysis

Three software artifacts (Fig. 2) have been developed for the analysis and visualization of strawberry architectural data and development: (i) the *OpenAlea.Strawberry* Python package, (ii) a set of interactive Jupyter Notebook tutorials, and (iii) an end-user web application. First, *OpenAlea.Strawberry* is an open-source Python package (https://github.com/openalea/strawberry), available in the OpenAlea platform (Pradal *et al*., 2008, 2015). *OpenAlea.Strawberry* relies mainly on Python 3, OpenAlea.PlantGL (Pradal, *et al*., 2009), and OpenAlea.MTG (Pradal and Godin, 2020). It provides methods for architecture reconstruction, analysis at the node and module scale, and 2D and 3D visualization. As shown in Fig. 2, it enables one to (i) load a MTG file format that encodes in a standard way (topology and property) the description of strawberry architectural data and provides a generic and multiscale MTG data structure; (ii) visualize in 2D and 3D architectural data of one plant (one or several genotypes), and (iii) analyse the architectural data at different scales (i.e. node, module, and plant scales) by providing various exploratory functions to plot and extract data for further analysis such as estimation of HHMSM models. 

Second, the Jupyter Notebook tutorials, designed for scientists, provide an interactive web interface to illustrate the various steps of the pipeline and run existing analyses in a reproducible way (Kluyver *et al*., 2016). The full software and user documentation, including some tutorials, is available and updated after each change (https://strawberry.rtfd.io). 

Finally, a web application has been designed on top of the *OpenAlea.Strawberry* package to automatically visualize and ­analyse strawberry architecture for end-users such as breeders. A demonstration of the application is provided through a video (Denoyes-Rothan and Pradal, 2022). This application is built using Voilà and Jupyter frameworks. The web application can be installed directly from github or using the Docker image (Merkel, 2014) available on DockerHub (https://hub.docker.com/r/openalea/strawberry) for faster deployment.

## Discussion

### An open-source software and modeling approach to characterize the complex architecture of strawberry

In strawberry as in other crop species, flower and fruit production is highly dependent on plant and inflorescence architecture (Eshed and Lippman, 2019). In addition to yield, the timing and duration of fruit production, which is also related to the architecture of the plant, is an essential trait for strawberry growers and breeders (Hytönen *et al*., 2008; Gaston *et al*., 2013; Perrotte *et al*., 2016; Tenreira *et al*., 2017; Andrés *et al*., 2021). Compared with most other crops, the specificity of strawberry is that the AXB apical meristem (AXM) can produce a branch (lateral or extension axis), a dormant bud (lateral non-extended axis), or a stolon (Savini *et al*., 2006; Costes *et al*., 2014; Fig. 4C). Furthermore, the fate of the AXM depends on its position on the axis, as demonstrated here (Fig. 3). An additional level of complexity in strawberry is that acquiring high-resolution plant architecture datasets requires a destructive step, namely dissecting the PC, which is a compact structure (Tenreira *et al*., 2017; Fig. 1). Powerful tools are thus required to analyse the large and complex datasets produced by phenotyping and to represent the evolution over time of 2D and 3D architectures of plant and inflorescence and their impact on flower and fruit production.

To address these challenges, we first developed a free and open-source software library and web application, called *OpenAlea.Strawberry*, that allows the analysis of phenotypic traits relevant to strawberry architecture, which include the occurrence of branching modules according to their order, as well as the complexity of inflorescences (Fig. 2). By combining 2D and 3D representation of strawberry plant development and exploratory statistical methods, our software enables comprehensive comparison of branching structure and inflorescence complexity in plants from diverse genotypes or under different environmental conditions. Using *OpenAlea.Strawberry*, we could analyse over time the phenotypic variation in plant and inflorescence architecture of six strawberry genotypes that were extensively described monthly at node scale. By considering the spatio-temporal growth of the plant and formally describing plants through MTGs (Godin and Caraglio, 1998), we were able to highlight and analyse the diversity of the growth strategies used by the various strawberry genotypes to build their architecture.

We then extracted from *OpenAlea.Strawberry* the data needed to build a model to identify homogeneous zones for axillary production (Fig. 3A). This model explicitly integrates the local scale of the phytomer and the more macroscopic scale of the branching zone, and allows for the study of spatial and temporal dependencies between different modules (Peyhardi *et al*., 2017). Using the HHMSM chain (Guédon, 2005; Guédon *et al*., 2022), we could show that the zeroth-order module (PC) is structured, regardless of genotype (except Gariguette), into three distinct and homogeneous zones with respect to AXM fate (Figs. 3A, B). The three zones produce either stolons and BCs (basal zone), or dormant buds (central zone), or BCs (proximal zone). In higher-order modules derived from the extension axis, AXM fate depends on order level, and the proportion of stolons increases with the module order (Fig. 3A). The increase in the proportion of stolons is likely due to long days (Kurokura *et al*., 2005; Messetani *et al*., 2011; Labadie *et al*., 2019) and/or low apical dominance (Neri *et al*., 2003). The original data produced by our analyses represent a significant advance over previous studies that did not consider the time scale or dependencies between different modules (Massetani *et al*., 2011; Bosc *et al*., 2012). They further allow comparison with *Rosaceae* tree species, for example apple and apricot, where the axis is structured in consecutive zones homogeneous in their axillary production (Renton *et al*., 2006; Costes *et al*., 2014; Mészáros *et al*., 2020). In strawberry, *OpenAlea.Strawberry* can be used to study the influence of the environment on the possible fluctuation of the three zones, as shown in apple (Mészáros *et al*., 2020).

### The date of occurrence of successive module orders has a major impact on flowering pattern

**Fig. 8.**
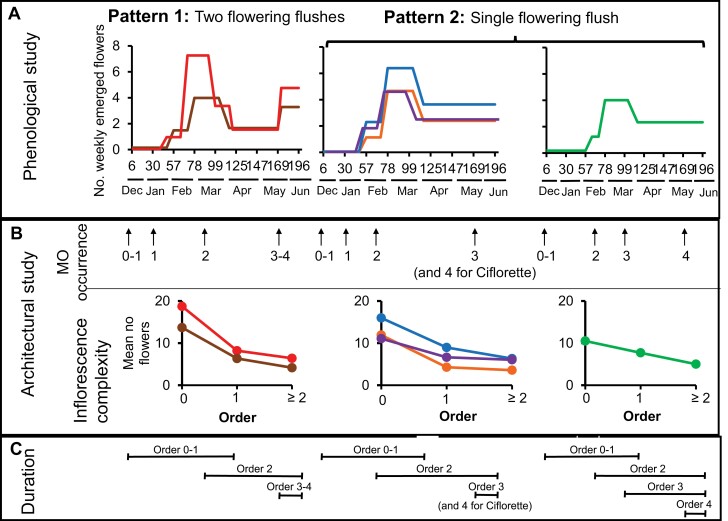
Relationships between flowering phenological phases and architectural data. (A) Phenological phases were identified using a longitudinal data modeling approach, which highlighted two patterns of flowering: two flushes separated by a phase of lower flower production (pattern 1) or a single flush (pattern 2). Flowering intensity was dependent on genotype. Calendar days and months are indicated in the *x*-axis. (B) Architectural study with module order occurrence identified by quantile 0.9 and inflorescence complexity (flower numbers per inflorescence according to the module order). (C) Duration of the flowering period according to the module order. Altogether, these results show that the intensity of flower production for the first flush is related to the complexity of the inflorescence at the zeroth and first-order modules. The presence of a near-stop of flowering in genotypes displaying pattern 1 can be explained by a delay in the occurrence of the second-order module. The absence of flowering stop after the first flush in genotypes displaying pattern 2 may be due either to the rapid relay of second-order modules for flowering, or to a higher production of lateral branch crowns.

The power of the approach describing the module orders at spatial and temporal levels (Peyhardi *et al*., 2017) was highlighted by the demonstration that the occurrence of module orders varies according to the genotype. Moreover, the time elapsed between the occurrence of two successive module orders can be more or less extended depending on the genotype (Table 1). By comparing the results from our temporal study of the architecture with the previous phenological analysis carried out on the same trial (Labadie *et al*., 2019), we could further link the occurrence and time elapsed between two successive module orders with the flowering patterns of each of the six genotypes (Fig. 8). In strawberry, the first flush of flowering is mainly produced by the zeroth- and first-order modules (Bosc *et al*., 2012; Massetani and Neri, 2016) (Fig. 8). These modules are initiated in the nursery when temperature and day length are reduced (Opstad *et al*., 2011; Heide *et al*., 2013). The resumption of flower production in May for Clery and Gariguette (Labadie *et al*., 2019) takes place approximately 2 months after the occurrence of the second-order modules (in March), which is consistent with a period of about 9 weeks between floral initiation and inflorescence emergence (Battey *et al*., 1998; Sønsteby and Heide, 2008). In the Capriss, Cir107, Ciflorette, and Darselect genotypes, the first flush of flowering is not followed by a stop in the appearance of flowers because the flowering of the second-order module takes over very quickly from that of the zeroth- and first-order modules. Our findings have considerable consequences for strawberry breeding, as shortening the intervals between the occurrence of module orders is critical for growers to control the yield and duration of fruit production (Kurokura *et al*., 2005). Indeed, a main goal of growers is to avoid the interruption of production.

### Genetic diversity in shoot branching is likely related to variability in apical dominance

In strawberry, shoot branching plays a central role in controlling fruit yield because each axis is terminated by an inflorescence when the environment is favorable (Kurokura *et al*., 2017; Tenreira *et al*., 2017; Gaston *et al*., 2020, 2021). Our 2D and 3D plant architecture representation (Fig. 4) indicated the great diversity for this trait among the various genotypes studied, which led to more or less bushy plants (Supplementary Figs S2–S4). Thanks to our HHMSM model, we could further show that branching occurs with very high probability in the very short distal zone (a single phytomer) and in the proximal zone. Branching is regulated by apical dominance, which exerts control over axillary bud outgrowth from the shoot apex (Cline, 1997; Barbier *et al*, 2017). Apical dominance is released when the SAM becomes floral or is removed, for example by grazing or pruning, thus allowing axillary buds to grow and new axes to form. In strawberry, an apex excision experiment (Neri *et al*., 2003) showed that lower AXBs, i.e. those corresponding to our basal zone, can develop into BCs when the buds are released from apical dominance. As previously suggested by Sugiyama *et al*. (2004) and observed in tomato (Lifschitz and Eshed, 2006), the genetic diversity observed among the six genotypes studied could be due to variability in the capacity of their axillary buds to be released from apical dominance. In the two bushy genotypes, Capriss and Cir107, the lower effect of dominance could lead to the specific fourth zone of branching observed in the basal part of the plant (Supplementary Fig. S7; Supplementary Table S11). These findings underline the importance of branching on the zeroth-order module for increasing the number of inflorescences of the plant. They further lay the foundation for breeding new strawberry cultivars with specific branching features adapted to the various patterns of fruit production required by the growers.

### The inflorescence complexity dictates the intensity of the first flowering flush

In addition to branching, inflorescence architecture is a key agronomical factor for yield (Eshed and Lippman, 2019; Gaarslev *et al*., 2021; Koppolu *et al*., 2022). In agreement with Guttridge (1955) and Ridout *et al*. (1999), we clearly show that the inflorescences of the zeroth-order module produced more flowers (Fig. 5) and therefore showed more complex inflorescences. We further demonstrate here that there is a sharp decrease of inflorescence complexity on the first-order module, followed by a slower reduction until stabilization in the higher-order modules (Fig. 5). Variations in the inflorescence complexity produced by the first two modules should therefore considerably affect the number of flowers produced by the plant. In agreement with previous findings (Darrow, 1929; Battey *et al*., 1998), we observed that the genetic background has a strong influence on inflorescence architecture. Moreover, of the six varieties studied, the two genotypes with the most complex inflorescences on the zero-order and first-order modules, Gariguette and Cir107, also exhibit the most intense first flush of flowering (Labadie *et al*., 2019) (Fig. 8). This trait is of the utmost importance to growers and constitutes a major breeding target for the control of fruit yield in strawberry. According to our results, breeding programs aimed at controlling the intensity of the first flowering flush should focus on the complexity of the first inflorescences. To translate flowering into fruit production, however, care should be taken to maintain the reproductive success of all flowers (Rindom and Hansen, 1995).

### Future directions

The genetic and molecular control of plant and inflorescence architecture has been deciphered in many domesticated crop species such as rice, maize, and tomato (Wang *et al*., 2006; Liu *et al*., 2013; Park *et al*., 2014a; Gaarslev *et al*., 2021). This knowledge has been widely exploited to increase crop yield (Eshed and Lippman, 2019), for example in tomato, a sympodial fruit-bearing species like strawberry (Gaston *et al*., 2020). In tomato, considerable increase in fruit yield has been achieved through genetic and biotechnological control of branching (Park *et al*., 2014b) and inflorescence complexity (Rodríguez-Leal *et al*., 2017). In strawberry, the plant architecture and thus the trade-off between fruit yield (through flowering) and daughter plant yield (through runners) depends on the fate of the AXM (Tenreira *et al*., 2017; Gaston *et al*., 2020, 2021). The subsequent difficulty of phenotyping and analysing plant architecture, in particular, has so far prevented progress similar to that in tomato, although significant advances have been made in our understanding of flowering regulation (Kurokura *et al*., 2017; Gaston *et al*., 2021) and AXM fate (Tenreira *et al*., 2017; Caruana *et al*., 2018; Andrés *et al*., 2021; Liang *et al*., 2022).

The free and open-source software library and web application provided here will allow users to analyse phenotypic traits related to strawberry architecture and yield, including the occurrence of modules and complexity of inflorescences. There are numerous directions for applied and basic research. Our tool and strategy described here will prove particularly useful to strawberry breeders for whom controlling yield pattern throughout the production period is essential. Future developments will, for example, use the data extracted from *OpenAlea.Strawberry* to predict the yield of a given strawberry variety based on its architecture and reproductive success. It will also be used to screen genetic resources by genome-wide association study and obtain information on the genetic control of phenotypic traits related to architecture, as well as to analyse the modulation of plant architecture by the environment, for example by light or temperature (Rantanen *et al*., 2014; Ye *et al*., 2021).

## Supplementary data

The following supplementary data are available at *JXB* online.

Fig. S1. Average temperature and global radiation at Douville in 2014 and 2015.

Fig. S2. 3D representation of the nine plants of the six genotypes.

Fig. S3. 3D representation of the nine plants without leaflets of the six genotypes.

Fig. S4. 2D schematic representation of the most central plant of the six genotypes during the seasonal production.

Fig. S5. Probabilities of the axillary production as a function of the node rank for the six varieties.

Fig. S6. Hidden hybrid Markov/semi-Markov chain in three homogeneous zones on the six genotypes for zeroth-order module.

Fig. S7. Hidden hybrid Markov/semi-Markov chain in four homogeneous zones on Capriss and Cir107 for zeroth-order module.

Table S1. Chilling requirement and flowering earliness for the six genotypes.

Table S2. Specification of the MTG for the cultivated strawberry.

Table S3. Characteristics of samples of sequences.

Table S4. Linear trend for the number of phytomers as function of module order for first-order and the number of flowers.

Table S5. Mean number of phytomers and grouping of genotypes using ANOVA.

Table S6. Mean number of flowers and grouping of genotypes using ANOVA.

Table S7. Mean number of stolons and grouping of genotypes using ANOVA.

Table S8. Module order frequency distribution for the six genotypes.

Table S9. Length and axillary production in the three zones identified by the HHMSM model.

Table S10. Probabilities of skipping proximal, median, or distal zones.

Table S11. Length and axillary production in the four zones identified by the HHMSM model.

Table S12. Zone length and axillary production of the first- to fourth- order modules.

Table S13. Probabilities of transition from a lateral branch crown in modules of order ≥1.

Table S14. Probabilities of transition leading to a stolon in modules of order ≥1.

erad097_suppl_Supplementary_Material

## Data Availability

All data are available at Github: https://github.com/openalea/strawberry/tree/master/share/data A demonstration video of plant architecture software "STRAWBERRY" is available at https://data.inrae.fr/dataset.xhtml?persistentId=doi:10.57745/XJY5RO; (Denoyes-Rothan and Pradal, 2022).

## Abbreviations

AXB: axillary bud
AXM: axillary meristem
BC: lateral branch crown
EC: extension crown
HHMSM: hidden hybrid Markov/semi-Markov
MTG: multiscale tree graph
PC: primary crown
SAM: shoot apical meristem
