# Supplementary material for: Spatio-temporal analysis of strawberry architecture: insights into the control of branching and inflorescence complexity
Source: J Exp Bot. 2023 Mar 26;74(12):3595–612. doi: 10.1093/jxb/erad097 (PMC10299788; doi:10.1093/jxb/erad097)
Supplement: erad097_suppl_Supplementary_Material [file erad097_suppl_supplementary_material.pdf]

SUPPLEMENTARY DATA for

Spatio-temporal analysis of strawberry architecture: insights into the control of branching and inflorescence complexity

Marc Labadie[1,2], **Karine Guy**[3], **Marie-Noëlle Demené**[3], **Yves Caraglio**[4], **Gaëtan Heidsieck**[1,2], **Amelia Gaston**[1], **Christophe Rothan**[1], **Yann Guédon**[2], **Christophe Pradal**[2,5*], and **Béatrice Denoyes**[1*]

[1]Univ. Bordeaux, INRAE, Biologie du Fruit et Pathologie, UMR 1332, F-33140, France

[2]CIRAD, UMR AGAP and Université de Montpellier, 34098 Montpellier, France

[3]INVENIO, MIN de Brienne, 110 quai de Paludate, 33800 Bordeaux, France

[4]CIRAD, UMR AMAP and Université de Montpellier, 34398 Montpellier, France

[5]Inria & LIRMM, Univ Montpellier, CNRS, Montpellier, France

♀Deceased

*Authors for correspondence:

Béatrice Denoyes; Email: beatrice.denoyes@inrae.fr

Christophe Pradal ; Email : christophe.pradal@cirad.fr

Supplementary Figures

Figure S1. Average temperature and global radiation at Douville in 2014 and 2015.

Figure S2. 3D representation of the 9 plants of the six genotypes during the seasonal production.

Figure S3. 3D representation of the 9 plants without leaflets of the six genotypes during the seasonal production.

Figure S4. 2D schematic representation of the most central plant of the six genotypes during the seasonal production.

Figure S5. Probabilities of the axillary productions as a function of the node rank for Gariguette, Ciflorette, Clery, Capriss, Darselect and Cir107.

Figure S6. Schematic representations of the Hidden hybrid Markov/semi-Markov chain in three homogeneous zones estimated on the six genotypes for zeroth-order module.

Figure S7. Schematic representations of the Hidden hybrid Markov/semi-Markov chain in four homogeneous zones estimated on Capriss and Cir107 for zeroth-order module.

Supplementary Tables

Table S1. Chilling requirement (in hours) and flowering earliness (with ordered categories early, median, late) for the six genotypes.

Table S2. Specification of the multiscale tree graph for the cultivated strawberry (botanical entity, scale and associated properties).

Table S3. Characteristics of samples of sequences: number of sequences, cumulative length of sequences, frequencies of modules for successive module orders, axillary productions in percentage.
Table S4. Linear trend (estimated slope and 95% confidence interval− IC 95 % −) for the number of phytomers as function of module order for first-order (order ≥ 1) onward and for the number of flowers as a function of module order for first-order (order ≥ 1) and second-order (order ≥ 2) onward.
Table S5. Mean number of phytomers with standard deviation (s.d.) and grouping of genotypes using ANOVA on ranks (Kruskal-Wallis test and associated post-hoc tests) represented by letters for zeroth-order module (order 0) and for modules of orders ≥ 1.
Table S6. Mean number of flowers with standard deviation (s.d.) and grouping of genotypes using ANOVA on ranks (Kruskal-Wallis test and associated post-hoc tests) represented by letters for zeroth-order module (order 0), first-order modules (order 1) and for modules of orders ≥ 2.
Table S7. Mean number of stolons with standard deviation (s.d.) and grouping of genotypes using ANOVA on ranks (Kruskal-Wallis test and associated post-hoc tests) represented by letters for successive module orders.
Table S8. Module order frequency distribution (with cumulative distribution function in parentheses) for the successive dates of observation for the six genotypes (Gariguette, Ciflorette, Clery, Capriss, Cir107 and Darselect).
Table S9. Length (no. phytomers) and axillary productions in percentage of the three zones, proximal, median and distal, identified by the HHMSM model built with a global model (pooled genotypes) on the zeroth-order module.
Table S10. Probabilities of skipping −proximal (prox.), median (med.) or distal (dist.)− zones in zeroth-order modules extracted from the segmentation in zones using a global model.
Table S11. Length (no. phytomers) and axillary productions in percentage of the four zones: supplementary, proximal, median and distal zones, identified by the HHMSM model for Capriss and Cir107 zeroth-order modules.
Table S12. Zone length (no.phytomeres) and axillary productions of the first- to fourth- order modules for the six genotypes.
Table S13. Probabilities of transition (and associated count in parentheses) from a lateral branch crown (BC: lateral branch crown; FB: floral bud; EC: extension crown) in modules of order ≥ 1.
Table S14. Probabilities of transition (and associated count in parentheses) leading to a stolon (AB: aborted bud; VB: vegetative bud; IB: initiated bud; FB: floral bud; ST: stolon) in modules of order ≥ 1.

Supplementary Video

https://data.inrae.fr/dataset.xhtml?persistentId=doi:10.57745/XJY5RO

Figure S1

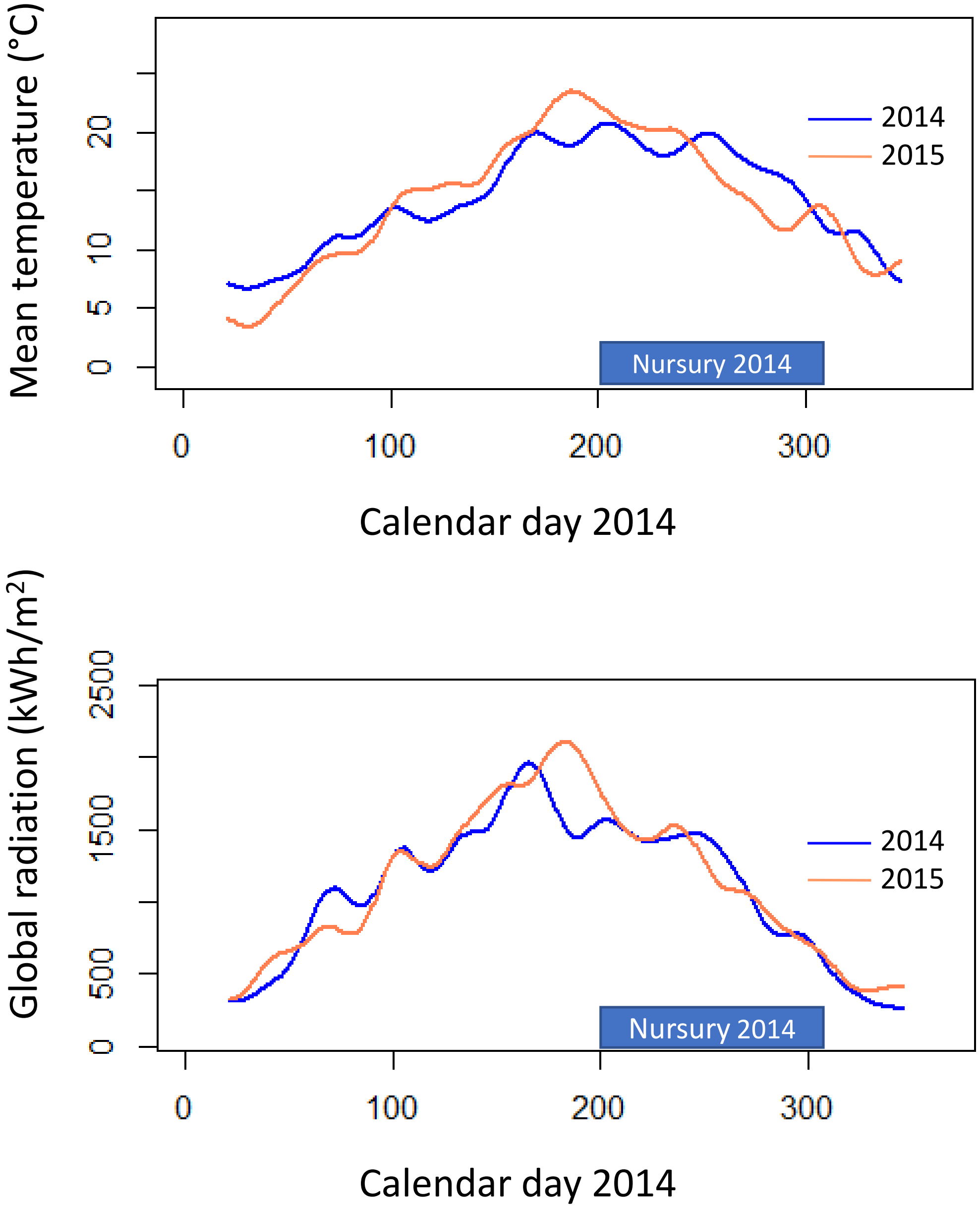

Figure S1. Smoothed curves of average temperature and global radiation at Douville in 2014 and 2015. In 2014, daughter plants were raised in the nursery starting in late July. They were placed in a climate chamber at 2°C on different dates in November to meet their chilling requirement (see Supplementary Table S1). Planting was done in December 2014 and the experiment was followed until mid-June 2015.

Figure S2

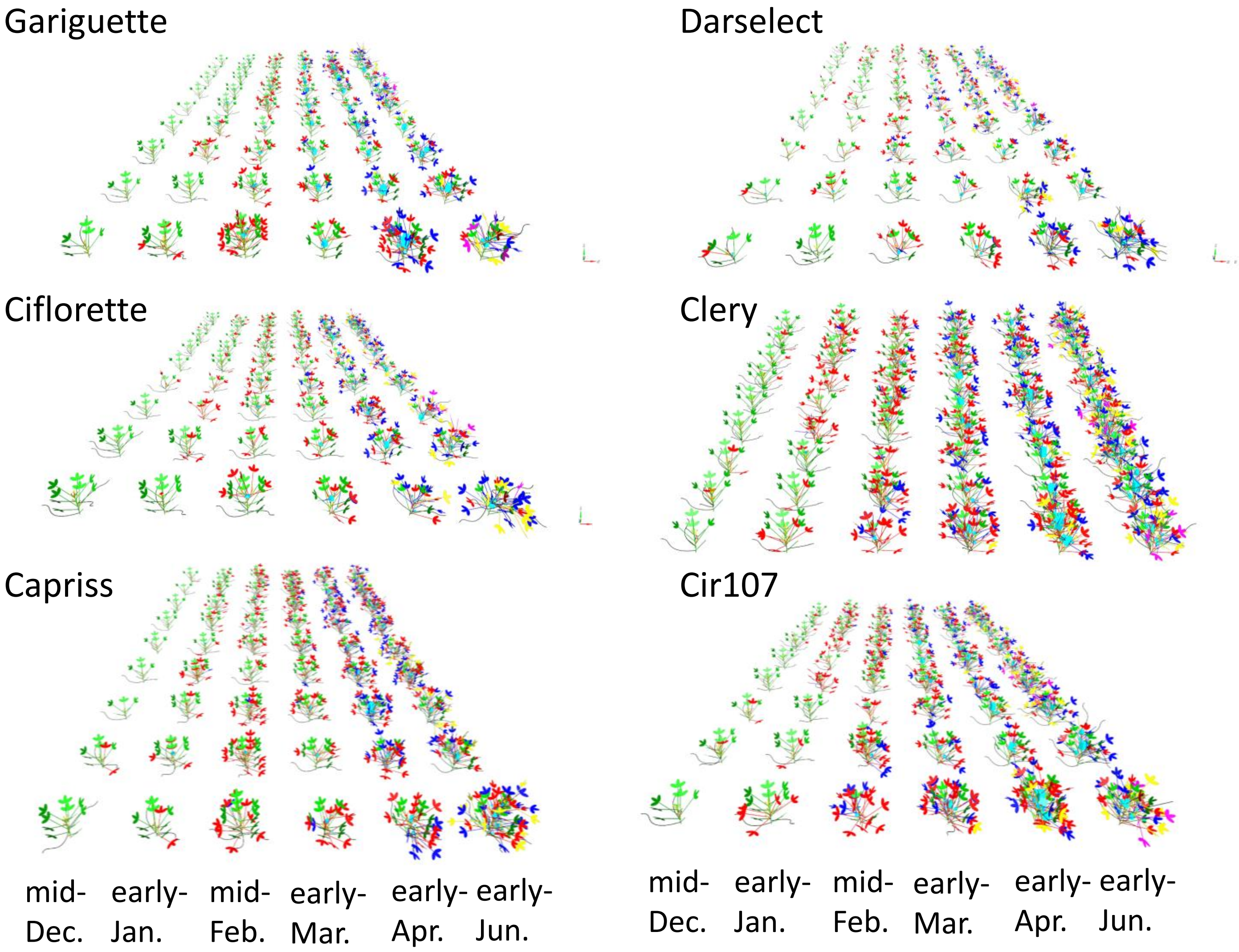

Figure S2. 3D representation of the nine plants of the six genotypes during the seasonal production. The seasonal production is represented by six successive dates of observation. Colors from green to purple represent module orders (green for zeroth-order, red for first-order, blue for second-order, yellow for third-order and purple for fourth-order). The size of the light blue boxes representing the inflorescence is proportional to the number of open flowers.

Figure S3

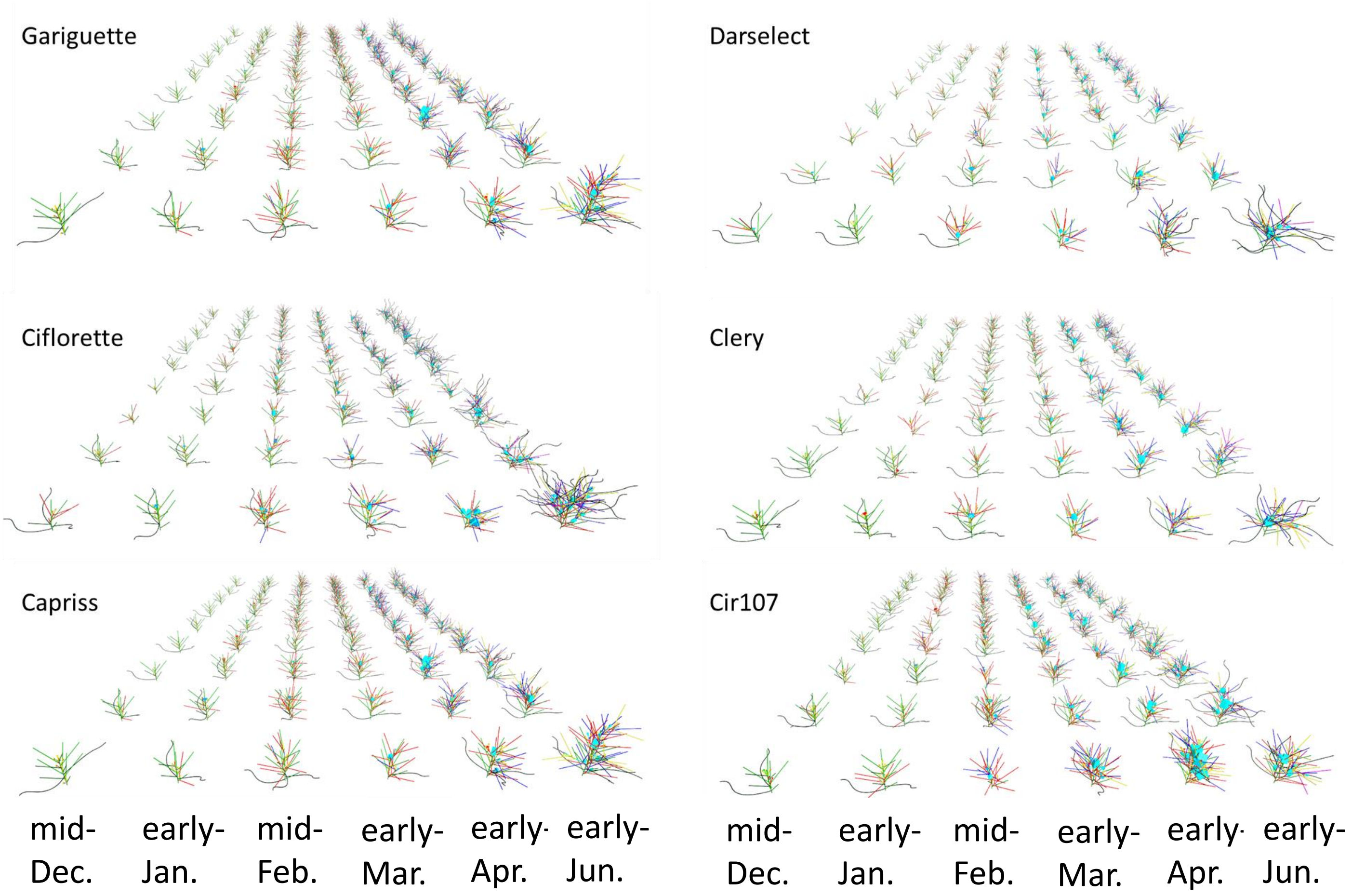

Figure S3. 3D representation of the nine plants without leaflets of the six genotypes during the seasonal production. The seasonal production is represented by six successive dates of observation. Colors from green to purple represent module orders (green for zeroth-order, red for first-order, blue for second-order, yellow for third-order and purple for fourth-order). The size of the light blue boxes representing the inflorescence is proportional to the number of open flowers.

Figure S4

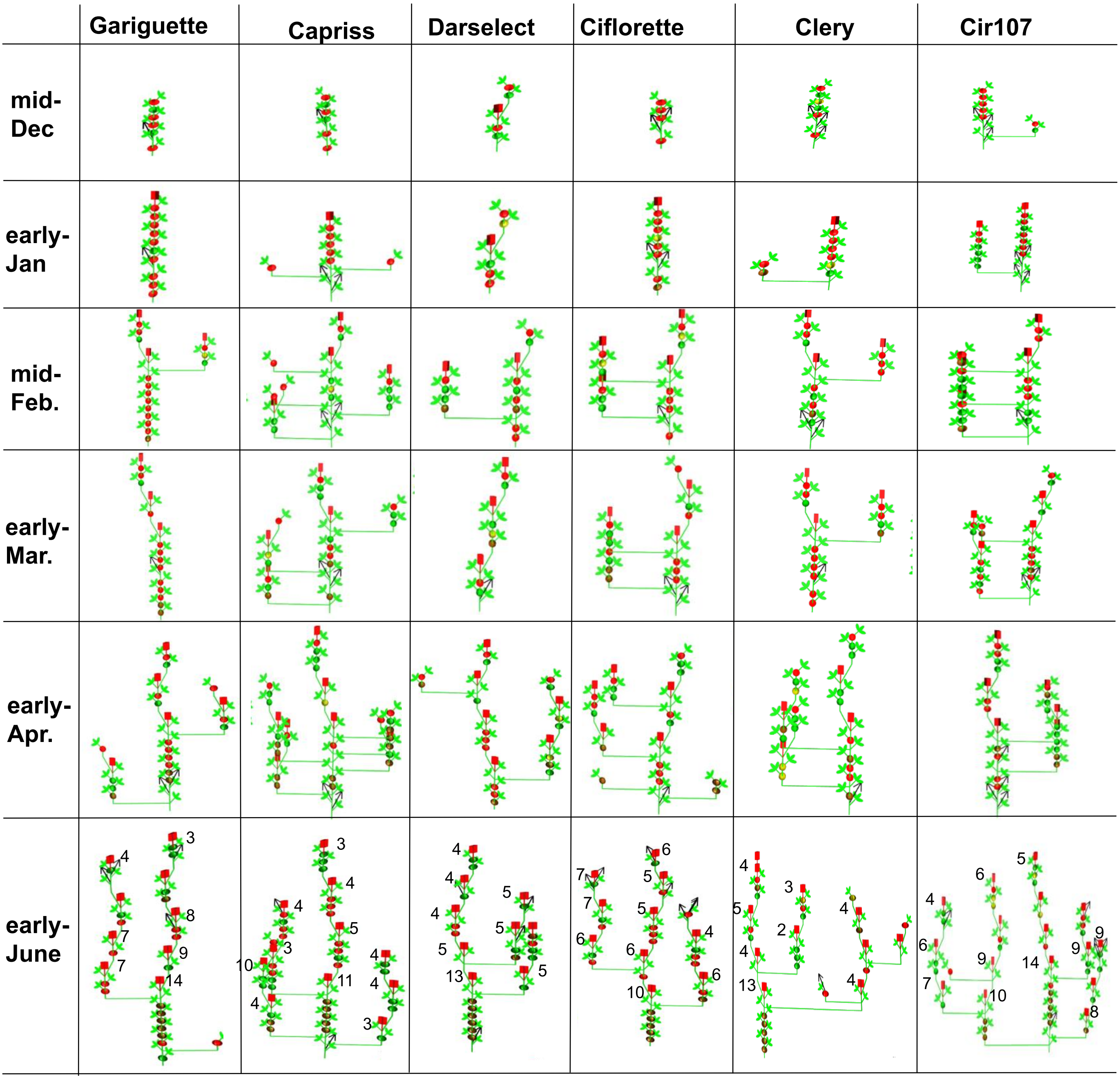

Figure S4: 2D schematic representation of the most central plant of the six genotypes during the seasonal production. The seasonal production is represented by six successive dates of observation. The spatio-temporal architecture of the six varieties is summarized by the representation of the most central individual for each date of observation. The representation of the development of a given genotype is discontinuous between two dates because the acquisition of the architecture require dissection and is destructive. At each date, a new plant of the same genotype is represented. Each organ is represented by a geometrical shape: inflorescence, red box; phytomer (internode + petiole + 3 leaflets), green cylinder terminated by three green discs; stolon, black arrow; axillary bud (AXB), a sphere colored according to the stage of the terminal meristem (green, yellow, red or brown for respectively vegetative, initiated, floral or aborted/dried stage). Lateral branch crown and extension crown were represented by horizontal or oblique shifts respectively. Apparent axes are materialized by the succession of extension modules from zeroth-order module or from a branch-crown. For the last date of observation (early June), the open flower number of each inflorescence was included. At this date, the dry dormant buds observed were considered as aborted buds. They were mainly located on the zero-order module.

Figure S5

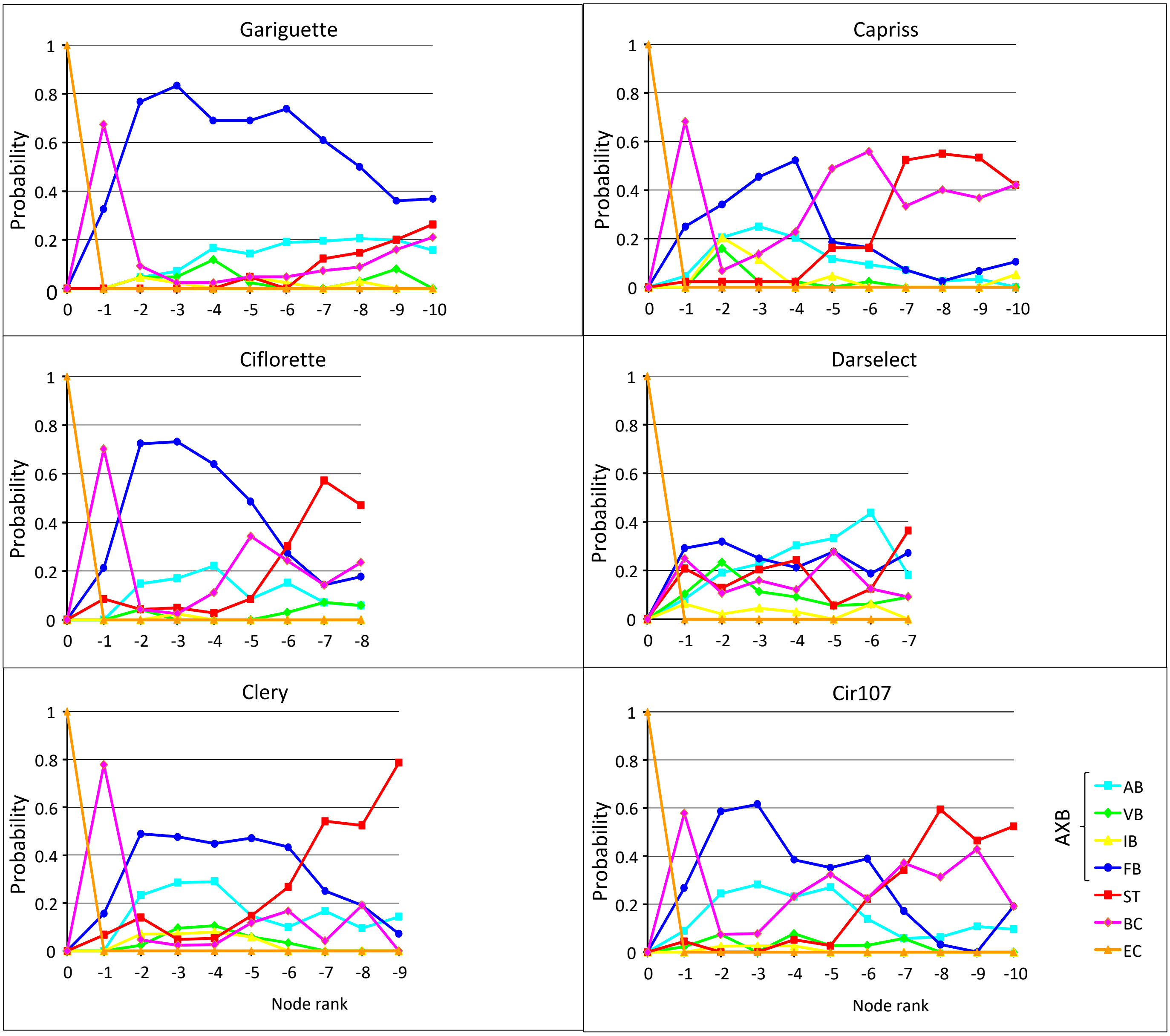

Figure S5: Probabilities of the axillary production as a function of the node rank for Gariguette, Ciflorette, Clery, Capriss, Darselect and Cir107. The node rank is ordered from the top to the base of the zeroth-order module without the last node bearing the extension crown. Axillary production was axillary bud (AXB) determined by the developmental stage of its AXM (AB: aborted bud, VB: vegetative bud, IB: initiated bud, FB: floral bud), stolon (ST), lateral branch crown (BC) or extension crown (EC).

Figure S6

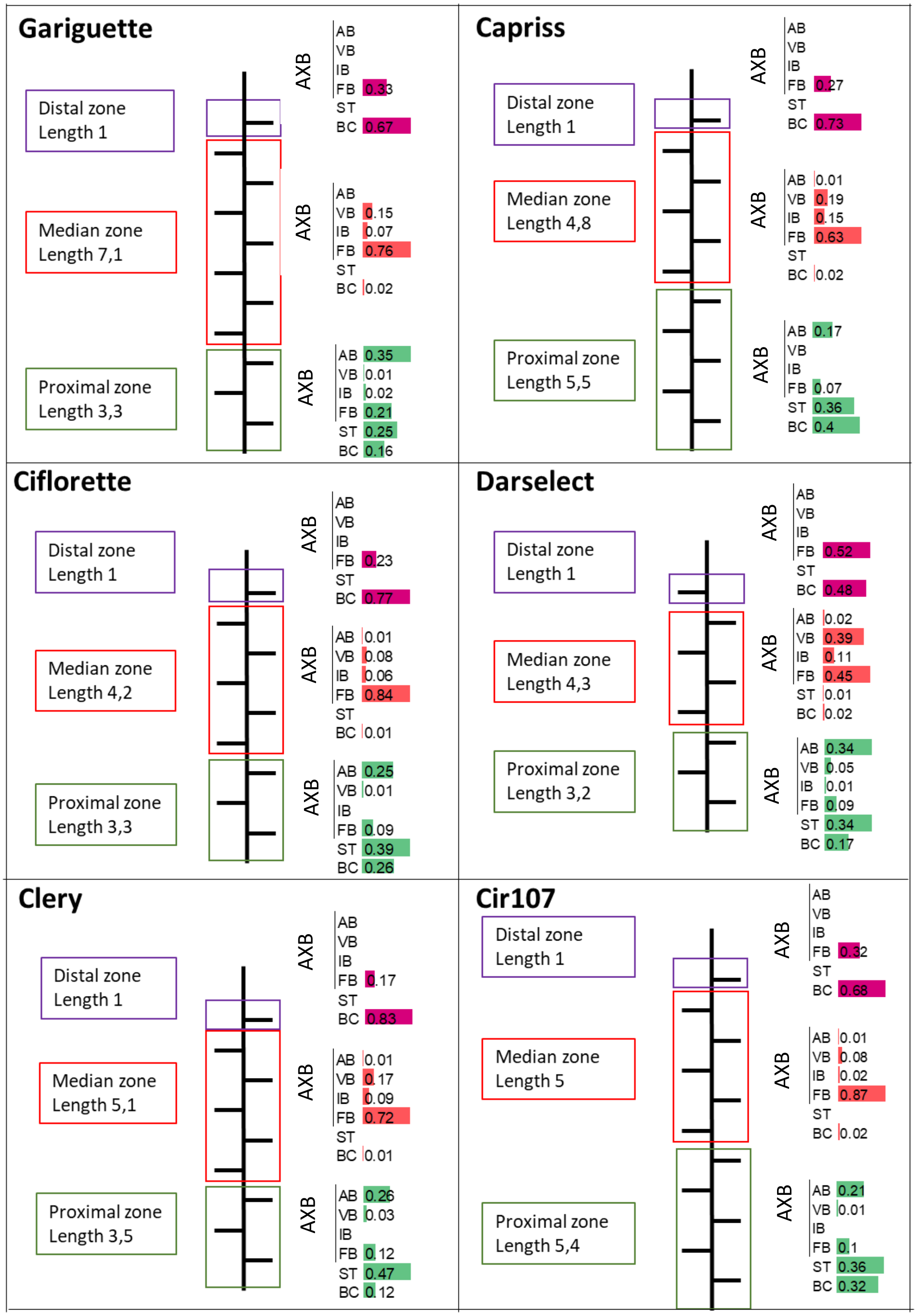

Figure S6. Schematic representations of the hidden hybrid Markov/semi-Markov chains in three homogeneous zones estimated on the six genotypes for zeroth-order module. Each zone is represented by a color box: green for proximal zone, red for median zone, and purple for distal zone. Each zone was characterized by the mean length zone (mean no. phytomers) and the non-generated observation distribution of axillary production (in percentage) colored in data bars. Axillary production was axillary bud (AXB) determined by the developmental stage of its AXM (AB: aborted bud, VB: vegetative bud, IB: initiated bud, FB: floral bud), stolon (ST) or lateral branch crown (BC).

Figure S7

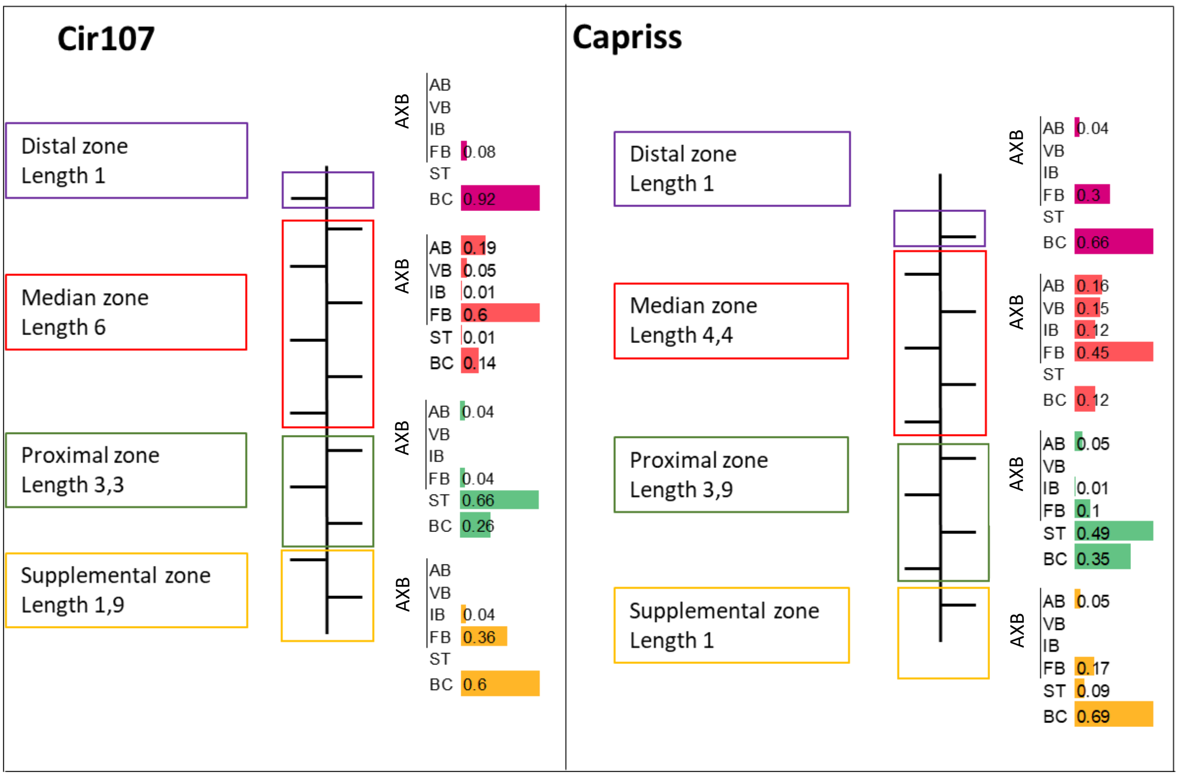

Figure S7: Schematic representations of the hidden hybrid Markov/semi-Markov chains in four homogeneous zones estimated on Capriss and Cir107 for zeroth-order module. Each zone is represented by a color box: green for proximal zone, red for median zone, and purple for distal zone. Each zone was characterized by the mean length zone (mean no. phytomers) and the non-generated observation distribution of axillary production (in percentage) indicated and colored in data bars. Axillary production was axillary bud (AXB) determined by the developmental stage of its AXM (AB: aborted bud, VB: vegetative bud, IB: initiated bud, FB: floral bud), stolon (ST) or lateral branch crown (BC).

Table S1. Chilling requirement (in hours) and flowering earliness (with ordered categories early, median, late) for the six genotypes.

	Chilling requirement	Entrance in climatic chamber for chilling requirement	Flowering earliness
Gariguette	800	6th Nov	early
Ciflorette	700	4th Nov	early
Clery	900	2nd Nov	median
Capriss	700	10th Nov	median
Cir107	300	27th Nov	median
Darselect	900	2nd Nov	late

Table S2. Specification of the multiscale tree graph for the cultivated strawberry (botanical entity, scale and associated properties).

Entity (symbol)	Scale (level)	Properties
Plant (P)	Plant (1)	Date, genotype, plant identifier
Primary crown (T)	Axis/module (2)	Diameter
Branch crown (A)	Axis/module (2)	Lateral branch crown or extension crown
Leaf primordium (f)	Organ (3)	–
Leaf (F)	Organ (3)	Petiole, central and left leaflet Lengths
Bud (bt)	Organ (3)	Stage of the meristem from 17 to A
Stolon (s)	Organ (3)	–
Inflorescence primordium (ht)	Organ (3)	Stage from B to H
Inflorescence (HT)	Organ (3)	Stage from I to 87 according to BBCH scale, total no. flowers, no. open flowers, no. aborted flowers

The axillary bud (dormant bud) is defined by its meristem stage, which was classed according to the ones described in Gaston *et al.*, 2021 (Supplemental Fig. 1): 17 or 19, the apical dome of the meristem is enclosed or partially enclosed in the developing stipule of the youngest leaf; A, Apical dome above the developing stipule; B, First bract primordium; C, Four distinct sepal primordia; D, Growth of sepal and petal. Adapted from Jahn and Dana (1970).

The BBCH scale is available at https://france2.wiki/wiki/BBCH-scale_(strawberry) and Meier et al., 1994.

Meier U, Graf H, Hack H, Hess M, Kennel W, Klose R, Mappes D, Seipp D, Stauss R, Streif J, van den Boom T. 1994. Phänologische Entwicklungsstadien des Kernobstes (Malus domestica Borkh. und Pyrus communis L.), des Steinobstes (Prunus-Arten), der Johannisbeere (Ribes-Arten) und der Erdbeere (Fragaria × ananassa Duch.). Nachrichtenbl. Dtsch. Pflanzenschutzd. 46:141-153.

Table S3. Characteristics of samples of sequences: number of sequences, cumulative length of sequences, frequencies of modules for successive module orders, axillary productions in percentage. Axillary productions were axillary buds (AXB) determined by the developmental stage of their AXM (AB: aborted bud, VB: vegetative bud, IB: initiated bud, FB: floral bud), stolon (ST) or lateral branch crown (BC). Letters represent difference between genotype using Pearson's chi-square test with a p-value p=0.05. Values with same letter are not significantly different.

			Order					Axillary production					
								AXB					
	No. sequences	Cumul. length	0	1	2	3	4	AB	VB	IB	FB	ST	BC
Gariguette	125	1182	54	94 a	62 a	21 a	9 a	0.1 a	0.27 a	0.07 a	0.43 a	0.06 a	0.07 a
Ciflorette	144	1228	54	115 ab	78 a	31 a	17 a	0.14 a	0.28 a	0.04 a	0.33 a	0.12 a	0.09 a
Clery	134	1111	54	98 a	63 a	34 a	14 a	0.16 a	0.27 a	0.06 a	0.29 a	0.13 a	0.09 a
Capriss	212	1707	54	190 c	102 b	31 a	4 a	0.15 a	0.41 a	0.06 a	0.19 a	0.08 a	0.11 a
Cir107	186	1594	54	154 bc	110 b	41 a	8 a	0.17 a	0.34 a	0.03 a	0.27 a	0.09 a	0.1 a
Darselect	107	909	54	87 a	57 a	39 a	8 a	0.18 a	0.38 a	0.05 a	0.22 a	0.1 a	0.07 a

Table S4. Linear trend (estimated slope and 95% confidence interval– IC 95 % –) for the number of phytomers as function of module order for first-order (order ≥ 1) onward and for the number of flowers as a function of module order for first-order (order ≥ 1) and second-order (order ≥ 2) onward.

	No. phytomers		No. flowers			
	Order ≥ 1		Order ≥ 1		Order ≥ 2	
	Slope	IC 95%	Slope	IC 95%	Slope	IC 95%
Gariguette	−0.12 ns	[−0.33, 0.1]	−0.99	[−1.49, −0.48]	−0.68 ns	[−1.36, 0]
Ciflorette	0.05 ns	[−0.08, 0.18]	−0.15 ns	[−0.44, 0.13]	0.33 ns	[−0.14, 0.79]
Clery	−0.05 ns	[−0.18, 0.08]	−1.01	[−1.34, −0.68]	−0.14 ns	[−0.58, 0.3]
Capriss	−0.06 ns	[−0.21, 0.1]	−0.51	[−0.75, −0.27]	−0.47 ns	[−0.97, 0.03]
Cir107	−0.24	[−0.43, −0.05]	−1.65	[−2.14, −1.17]	−1.13	[−1.73, −0.53]
Darselect	−0.3	[−0.46, −0.14]	−1.26	[−1.69, −0.82]	−0.22 ns	[−0.59, 0.15]

ns, indicates that the slope is non-significantly different from zero.

Table S5. Mean number of phytomers with standard deviation (s.d.) and grouping of genotypes using ANOVA on ranks (Kruskal-Wallis test and associated post-hoc tests) represented by letters for zeroth-order module (order 0) and for modules of orders ≥ 1.

	Order 0			Order ≥ 1		
	Mean	s.d.	Group	Mean	s.d.	Group
Gariguette	10.43	2.35	a	3.33	1.31	bc
Ciflorette	7.63	2.57	b	3.4	1.01	ab
Clery	8.33	2.97	b	3.16	0.91	bc
Capriss	10.35	1.82	a	3.51	1.02	a
Cir107	9.63	3.29	a	3.43	1.36	ab
Darselect	6.11	2.29	c	3.02	1.07	c

Values in a column followed by different letters are different at $P = 0.05$.

Table S6. Mean number of flowers with standard deviation (s.d.) and grouping of genotypes using ANOVA on ranks (Kruskal-Wallis test and associated post-hoc tests) represented by letters for zeroth-order module (order 0), first-order modules (order 1) and for modules of orders ≥ 2.

	Order 0			Order 1			Order ≥ 2		
	Mean	s.d.	Group	Mean	s.d.	Group	Mean	s.d.	Group
Gariguette	18.74	3.68	a	8.2	3.25	ab	6.4	2.15	a
Ciflorette	11.08	2.19	d	6.64	2.07	c	6.06	1.68	a
Clery	13.71	3.34	c	6.34	2.13	c	4.17	1.27	c
Capriss	11.92	2.87	d	4.28	1.28	d	3.56	1.11	c
Cir107	16	4.2	b	8.98	3.46	a	6.32	1.87	a
Darselect	10.5	4.77	d	7.69	3.03	b	5	1.12	b

Values in a column followed by different letters are different at $P = 0.05$.

Table S7. Mean number of stolons with standard deviation (s.d.) and grouping of genotypes using ANOVA on ranks (Kruskal-Wallis test and associated post-hoc tests) represented by letters for successive module orders.

	Order 0			Order 1			Order 2			Order 3			Order 4		
	Mean	s.d.	Group	Mean	s.d.	Group	Mean	s.d.	Group	Mean	s.d.	Group	Mean	s.d.	Group
Gariguette	0	0	c	0	0	a	0.1	0.65	a	0.29	0.56	b	1.2	0.79	ab
Ciflorette	1.09	0.96	bc	0	0	a	0	0	a	0.87	0.85	a	1.8	0.77	a
Clery	1.65	1.25	ab	0.01	0.10	a	0.1	0.35	a	0.38	0.70	b	0.79	0.70	b
Capriss	1.96	0.97	a	0	0	a	0.01	0.10	a	0.2	0.41	b			
Cir107	1.83	1.33	a	0.03	0.25	a	0	0	a	0.29	0.56	b	1	0.76	ab
Darselect	0.94	1.11	c	0.01	0.11	a	0.09	0.34	a	0.23	0.48	b	0.67	1	b

Values in a column followed by different letters are different at $P = 0.05$. Empty cells: For Capriss, fourth-order modules do not exist.

Table S8. Module order frequency distribution (with cumulative distribution function in parentheses) for the successive dates of observation for the six genotypes (Gariguette, Ciflorette, Clery, Capriss, Cir107 and Darselect). The cumulative frequencies for each order and each date of observation are given respectively in the last column and row.

	Order	mid-Dec.	early Jan.	mid-Feb.	early Mar.	early Apr.	early June	Frequency
Gariguette	0	9 (1)	9 (0.56)	9 (0.27)	9 (0.24)	9 (0.14)	9 (0.11)	54
	1		7 (1)	24 (1)	17 (0.68)	26 (0.53)	20 (0.36)	94
	2				11 (0.97)	28 (0.95)	23 (0.65)	62
	3				1(1)	3 (1)	17 (0.87)	21
	4						9 (0.99)	9
	5						1 (1)	1
	Frequency	9	16	33	38	66	79	241
Ciflorette	0	9 (0.69)	9 (0.64)	9 (0.2)	9 (0.18)	9 (0.14)	9 (0.09)	54
	1	4 (1)	5 (1)	30 (0.85)	25 (0.68)	25 (0.52)	24 (0.31)	113
	2			7 (1)	15 (0.98)	29 (0.96)	27 (0.56)	78
	3				1 (1)	3 (1)	27 (0.81)	31
	4						17 (0.97)	17
	5						3 (1)	3
	Frequency	13	14	46	50	66	107	296
Clery	0	9 (0.82)	9 (0.47)	9 (0.31)	9 (0.24)	9 (0.14)	9 (0.09)	54
	1	2 (1)	10 (1)	20 (1)	21 (0.79)	24 (0.51)	21 (0.29)	98
	2				7 (0.97)	27 (0.92)	29 (0.58)	63
	3				1 (1)	5 (1)	28 (0.86)	34
	4						14 (1)	14
	Frequency	11	19	29	38	65	81	263
Capriss	0	9 (0.8)	9 (0.26)	9 (0.17)	9 (0.14)	9 (0.1)	9 (0.07)	54
	1	2 (1)	24 (0.97)	38 (0.87)	39 (0.74)	44(0.58)	43 (0.41)	190
	2		1 (1)	7 (1)	17 (1)	37 (0.99)	40 (0.73)	102
	3					1 (1)	30 (0.97)	31
	4						4 (1)	4
	Frequency	11	33	54	65	91	126	380
Cir107	0	9 (0.64)	9 (0.31)	9 (0.16)	9 (0.13)	9 (0.12)	9 (0.08)	54
	1	5 (1)	17 (0.9)	33 (0.74)	33 (0.58)	31 (0.52)	35 (0.38)	154
	2		3 (1)	14 (0.98)	28 (0.96)	30 (0.91)	35 (0.68)	110
	3			1 (1)	3 (1)	7 (1)	30 (0.93)	41
	4						8 (1)	8
	Frequency	14	29	57	73	77	117	367
Darselect	0	9 (0.6)	9 (0.45)	9 (0.31)	9 (0.24)	9 (0.14)	9 (0.12)	54
	1	6 (1)	11 (1)	17 (0.9)	15 (0.63)	20 (0.44)	18 (0.35)	87
	2			3 (1)	10 (0.89)	20 (0.74)	24 (0.66)	57
	3				4 (1)	17 (1)	18 (0.89)	39
	4						8 (0.9)	8
	5						1 (1)	1
	Frequency	15	20	29	38	66	78	246

Table S9. Length (no. phytomers) and axillary productions in percentage of the three zones, proximal, median and distal, identified by the HHMSM model built with a global model (pooled genotypes) on the zeroth-order module. Axillary productions were axillary buds (AXB) determined by the developmental stage of their AXM (AB: aborted bud, VB: vegetative bud, IB: initiated bud, FB: floral bud), stolon (ST) or lateral branch crown (BC).

	Proximal zone							Median zone							Distal zone						
		Axillary productions							Axillary productions							Axillary productions					
	Mean	AXB				ST	BC	Mean	AXB				ST	BC	Mean	AXB				ST	BC
	length	AB	VB	IB	FB			length	AB	VB	IB	FB			length	AB	VB	IB	FB		
Gariguette	3.3	0.35	0.01	0.02	0.21	0.25	0.16	7.1		0.15	0.07	0.76		0.02	1				0.33		0.67
Ciflorette	3.4	0.25	0.01		0.09	0.39	0.26	4.2	0.01	0.08	0.06	0.84		0.01	1				0.23		0.77
Clery	3.5	0.26	0.03		0.12	0.47	0.12	5.1	0.01	0.17	0.09	0.72		0.01	1				0.17		0.83
Capriss	5.5	0.17			0.07	0.36	0.4	4.8	0.01	0.19	0.15	0.63		0.02	1				0.27		0.73
Cir107	5.4	0.21	0.01		0.1	0.36	0.32	5	0.01	0.08	0.02	0.87		0.02	1				0.32		0.68
Darselect	3.2	0.34	0.05	0.01	0.09	0.34	0.17	4.3	0.02	0.39	0.11	0.45	0.01	0.02	1				0.52		0.48

Empty cells: the probability is zero.

Table S10. Probabilities of skipping −proximal (prox.), median (med.) or distal (dist.)− zones in zeroth-order modules extracted from the segmentation in zones using a global model.

	Prox.	Prox. + med.	Med.	Dist.	Med. + dist.
Gariguette	0.22				0.05
Ciflorette	0.17		0.16		0.09
Clery	0.02	0.04	0.25		0.06
Capriss			0.17		0.06
Cir107		0.07	0.1		0.16
Darselect	0.15		0.15	0.25	0.37

Empty cells: the probability is zero.

Table S11. Length (no. phytomers) and axillary productions in percentage of the four zones: supplementary, proximal, median and distal zones, identified by the HHMSM model for Capriss and Cir107 zeroth-order modules. Axillary productions were axillary buds (AXB) determined by the developmental stage of their AXM (AB: aborted bud, VB: vegetative bud, IB: initiated bud, FB: floral bud), stolon (ST) or lateral branch crown (BC).

	Supplementary zone							Proximal zone						
	Mean length	Axillary productions						Mean length	Axillary productions					
		AXB				ST	BC		AXB				ST	BC
		AB	VB	IB	FB				AB	VB	IB	FB		
Capriss	1	0.05	0	0	0.17	0.09	0.69	3.9	0.05	0	0.01	0.1	0.49	0.35
Cir107	1.9	0	0	0.04	0.36	0	0.6	3.3	0.04	0	0	0.04	0.66	0.26

	Median zone							Distal zone						
	Mean length	Axillary productions						Mean length	Axillary productions					
		AXB				ST	BC		AXB				ST	BC
		AB	VB	IB	FB				AB	VB	IB	FB		
Capriss	4.4	0.16	0.15	0.12	0.45	0	0.12	1	0.04	0	0	0.3	0	0.66
Cir107	6	0.19	0.05	0.01	0.6	0.01	0.14	1	0	0	0	0.08	0	0.92

Table S12. Zone length (no.phytomeres) and axillary productions in percentage of the first- to fourth- order modules for the six genotypes. Axillary productions were axillary buds (AXB) determined by the developmental stage of their AXM (AB: aborted bud, VB: vegetative bud, IB: initiated bud, FB: floral bud), stolon (ST) or lateral branch crown (BC).

	first-order							second-order						
		Axillary productions							Axillary productions					
		AXB				ST	BC		AXB				ST	BC
	Mean length	AB	VB	IB	FB			Mean length	AB	VB	IB	FB		
Gariguette	2.63	0.08	0.46	0.10	0.31		0.05	4.04	0.15	0.49	0.09	0.23	0.03	0.01
Ciflorette	2.53	0.20	0.40	0.06	0.29		0.05	3.57	0.11	0.52	0.04	0.32		0.01
Clery	2.49	0.24	0.36	0.09	0.25		0.06	3.04	0.11	0.51	0.07	0.25	0.03	0.03
Capriss	3.11	0.20	0.61	0.07	0.11		0.01	4.32	0.14	0.60	0.06	0.19		
Cir107	3.35	0.20	0.47	0.04	0.25	0.01	0.03	3.51	0.18	0.59	0.04	0.18		0.02
Darselect	2.91	0.23	0.47	0.06	0.20		0.04	2.08	0.08	0.54	0.04	0.24	0.04	0.04

	third-order							fourth-order						
		Axillary productions							Axillary productions					
		AXB				ST	BC		AXB				ST	BC
	Mean length	AB	VB	IB	FB	ST	BC	Mean length	AB	VB	IB	FB		
Gariguette	2.13	0.19	0.37	0.05	0.26	0.14		1.78		0.37			0.62	
Ciflorette	2.05	0.06	0.29	0.08	0.14	0.43		3.72	0.25	0.04	0.02	0.02	0.67	
Clery	2.32	0.14	0.39	0.07	0.19	0.18	0.04	2.69	0.32	0.29	0.03		0.35	
Capriss	2.51	0.17	0.59	0.06	0.10	0.08		3.54	0.62	0.00	0.25		0.12	
Cir107	2.70	0.15	0.53	0.06	0.14	0.11		4.30	0.28	0.32	0.08		0.32	
Darselect	3.56	0.10	0.58	0.05	0.14	0.12	0.01	3.63	0.39	0.28	0.06		0.28	

Empty cells: the probability is zero.

Table S13. Probabilities of transition (and associated count in parentheses) from a lateral branch crown (BC: lateral branch crown; FB: floral bud; EC: extension crown) in modules of order ≥ 1.

	P(BC → FB)	P(BC → BC)	P(BC → EC)
Gariguette			1 (14)
Ciflorette	0.07 (1)		0.93 (14)
Clery	0.05 (1)		0.95 (21)
Capriss			1 (4)
Cir107	0.18 (3)	0.12 (2)	0.7 (12)
Darselect			1 (14)

Empty cells: the probability is zero.

Table S14. Probabilities of transition (and associated count in parentheses) leading to a stolon (AB: aborted bud; VB: vegetative bud; IB: initiated bud; FB: floral bud; ST: stolon) in modules of order ≥ 1.

	P(AB → ST)	P(VB → ST)	P(IB → ST)	P(FB → ST)	P(ST → ST)
Gariguette	0.02 (1)				0.36 (8)
Ciflorette					0.39 (23)
Clery	0.03 (3)				0.23 (7)
Capriss	0.01 (1)				
Cir107	0.03 (4)	0.01 (3)			0.25 (6)
Darselect		0.02 (4)	0.05 (1)	0.02 (1)	0.2 (4)

Empty cells: the probability is zero.
